# Efficient pathogen screening in honey bees: Application of FTA^®^ cards for DNA storage and PCR analysis

**DOI:** 10.1371/journal.pone.0334066

**Published:** 2025-10-30

**Authors:** Kristýna Myslínová, Silvie Dostálková, Jana Jemelková, Beata Hurychová, Ondřej Biemann, Jan Brus, Jana Fürstová, Marek Petřivalský, Jiří Danihlík

**Affiliations:** 1 Department of Biochemistry, Faculty of Science, Palacký University Olomouc, Olomouc, Czech Republic; 2 Department of Cell Biology and Genetics, Faculty of Science, Palacký University Olomouc, Olomouc, Czech Republic; 3 Department of Geoinformatics, Faculty of Science, Palacký University Olomouc, Olomouc, Czech Republic; 4 Olomouc University Social Health Institute (OUSHI), Palacký University Olomouc, Olomouc, Czech Republic; University of Kwazulu-Natal, SOUTH AFRICA

## Abstract

Screening honey bee pathogens is crucial for early infection detection, which helps prevent pathogen transmission. The most widely used method for pathogen detection in honey bees is polymerase chain reaction (PCR). FTA^®^ cards (Qiagen) were compared with DNA-isolation kit DNeasy Plant Mini kit (Qiagen) for the detection of selected pathogens in honey bee samples collected from colonies in the Czech Republic in autumn and spring. FTA^®^ cards provide highly accurate results for detecting *N. ceranae* with a sensitivity of 97.2% and a specificity of 100%. Thus, FTA^®^ cards represent a reliable and cost-effective alternative to traditional methods for *N. ceranae* detection. Seasonal variation in pathogen prevalence was also assessed using FTA^®^ cards, revealing significant differences between autumn and spring. In total 85 samples were analysed for main bee pathogens (*N. ceranae*, *Nosema apis*, *Lotmaria passim*, *Crithidia mellificae,* and *Serratia marcescens*). Greater diversity pathogen occurence was observed in autumn, with 32% of colonies showing no detectable levels of the tested pathogens, 48% infected by one pathogen, 16% by two, and 4% by three; whereas in spring, 40% of colonies tested negative for all target pathogens, with 51% infected by one pathogen and 9% by two. In autumn 2020, *S. marcescens* was the most prevalent pathogen (46%), followed by *N. ceranae* (28%) and *L. passim* (18%), while no *C. mellificae* or *N. apis* were detected. In spring 2021, *N. ceranae* dominated with a 60% prevalence, and other pathogens were detected in only one sample each. FTA^®^ were found to be a more economical and faster alternative to commercial DNA isolation kits, particularly for *N. ceranae*. Moreover, FTA® cards maintained DNA stability under challenging conditions, including high temperatures, UV radiation, and oxidative stress, making them highly suitable for field applications. Collecting field samples on FTA^®^ cards preserves DNA integrity and mitigates degradation risks associated with improper shipment of whole bees.

## Introduction

Honey bee health is continuously threatened by pathogens that affect both adult bees and brood, including viruses, bacteria, fungi, and mites. Both adult bees and brood encounter a diverse array of microbial pathogens, resulting in a complex network of diseases [1,2]. Pathogens spread rapidly within densely populated hives, particularly under stress conditions from poor forage availability, or pesticide exposure. Infections may weaken individual bees, reduce colony size, deplete honey reserves, and impair thermoregulation, potentially culminating in winter colony collapse [3].

Certain pathogens, such as *Melissococcus plutonius* or *Paenibacillus larvae*, produce clear clinical symptoms in larvae. However, many infections in adult bees remain asymptomatic or manifest only when pathogen loads are high or additional stressors are present. Consequently, laboratory diagnostics are essential for the accurate detection and monitoring of colony health [1,4]. This study focuses on intestinal pathogens of adult bees that are commonly monitored during honey bee health assessments: *Nosema apis*, *Nosema ceranae*, *Serratia marcescens*, *Crithidia mellificae*, and *Lotmaria passim*. Although these microorganisms differ in origin and biology, they all compromise bee health by disrupting the gut environment, reducing lifespan, and potentially interacting synergetically [5–8]. Coinfections are common and can exacerbate the effects of individual pathogens, as observed, for instance, between *Nosema* spp. and *S. marcescens* [9], or with trypanosomatids [10,11].

Honey bee pathogen prevalence varies according to region and season and is influenced by local conditions and beekeeping practices [12]. Regular monitoring is essential for tracking infections and managing their impact on bee health. Detection methods include light microscopy, which enables the identification of *Nosema spp.* spores but fails to differentiate reliably between species [13]. Microbial cultivation can identify some pathogens like *S. marcescens*, which forms red colonies on agar, but it is less effective for *Nosema* and protozoa [14,15]. PCR remains a key method for specific detection, allowing multiplex assays for multiple pathogens [16,17]. However, PCR is costly due to the expense of DNA isolation kits [18]. A potential alternative, FTA^®^ cards, immobilise nucleic acids for long-term, room-temperature storage, making them compact and ideal for transport [19]. They enable easy sampling for PCR without extensive processing, lowering cost and complexity [20,21].

Evans, Schwarz [18], in the COLOSS BEEBOOK outlining standardised methods and protocols for honey bee research, argue that FTA^®^ cards are unsuitable for adult bees and bulk samples due to uneven sample distribution and high associated costs. To address these limitations, our study aims to refine and adapt FTA^®^ card protocols to be specifically suited for honey bee pathogen research. Furthermore, BEEBOOK’s “Standard methods for *Nosema* research” uses a lengthy sample preparation protocol in order to lyse *Nosema spp.* spores, followed by DNeasy® Plant Mini kit (Qiagen) protocol [13]. *Nosema spp.*, compared to other pathogens, has a rigid cell wall, which makes the sample processing step longer. This process could potentially be streamlined by using FTA^®^ cards. This would enable detection of multiple pathogens from a single, uniformly processed sample.

This study highlights the importance of rapid, accurate, and cost-effective detection of honey bee pathogens, particularly for epidemiological and ecological research. Traditional molecular diagnostics, though effective, are costly and time-consuming, especially at the point of DNA isolation and purification, prompting the need for streamlined methods. This study aimed to optimise DNA isolation from honey bee samples using FTA^®^ cards, enhancing the speed and sensitivity of pathogen detection. Additionally, pathogen prevalence was mapped across the Czech Republic, focusing on *S. marcescens*, *N. ceranae, N. apis*, *C. mellificae*, and *L. passim*. These data contribute to a deeper understanding of honey bee disease ecology by revealing regional and seasonal patterns, offering a foundation for future research on pathogen-host dynamics.

## Materials and methods

### Sample collection and processing

The samples were voluntarily provided by 57 beekeepers from various regions of the Czech Republic. Adult worker bees (not differentiated by age) were collected from individual hives (each from a separate apiary) in the autumn of 2020 (October) and then subsequently from the same hives in the spring of 2021 (April). Approximately 50 live bees were collected by each beekeeper from the experimental colony and immediately euthanised by freezing. Subsequently, bees placed in small paper boxes (approximately the size of a matchbox) were sent to the laboratory and stored at −20 °C. From autumn 2020, 50 samples were analysed. Due to the poor condition of some of the samples sent (e.g., mould on the bees) or colony mortality, only 35 samples from spring 2021 were used for analysis. In total, seven beekeepers failed to submit samples in autumn 2020 and were therefore excluded from the study.

Bioreba extraction bags (BIOREBA AG, Switzerland) were used for homogenisation of whole honey bees. A sample of 20 bees from each tested colony, as recommended by Pirk, de Miranda [22], were placed into one-half of the extraction bags. Three ml of water were added, and the bags were sealed by lamination. Using a pestle, the samples within the bags were initially roughly homogenised by pounding. The bags were then placed in a MiniMix^®^CC homogeniser (Interscience, France) for 1 minute at speed 4. This homogenisation step was repeated once. The bags were then cut open, and approximately 2 ml of liquid homogenates were pipetted into microtubes and frozen at −20 °C until further processing.

### Microscopic detection of *Nosema spp.*

As a control for subsequent PCR detection of *N. apis* and *N. ceranae*, light microscopy was used to detect *Nosema* spp spores. Sample homogenates (in total 85) were observed under a microscope (Olympus BX50, Olympus, Japan) to evaluate the presence of spores using 400 × magnification.

### DNA extraction

#### DNeasy^®^ Plant Mini kit (Qiagen).

A starting material of 90 µl of bee homogenates was used for DNA isolation, following the manufacturer’s protocol with the exception of omitting the RNA degradation step. The obtained samples had a consistent concentration of approximately ± 30 ng DNA/µl.

#### QIAcard^®^ FTA^®^ Classic (Qiagen).

**Fig 1:**
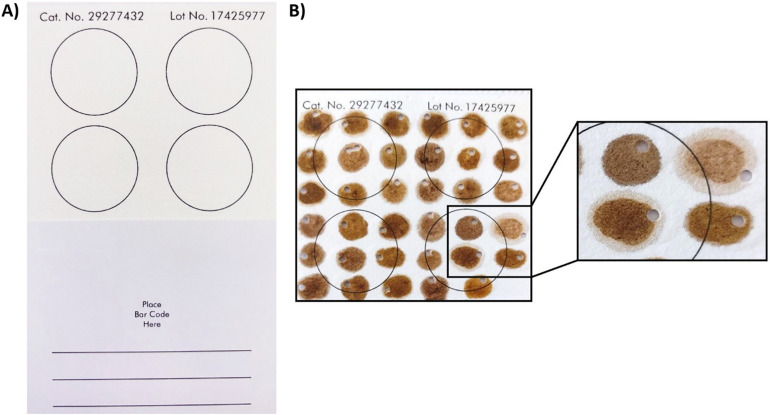
A) Clean FTA^®^ card. B) Application of honey bee homogenates on a FTA^®^ card. Total 36 samples were applied in 6 rows and 6 columns, every sample covering a circle with a diameter of approximately 1 cm.

The cards are marked withfour printed circles indicating sample placement (see Fig 1A). However, the whole area of the card can be used for storage of a higher number of samples. Therefore, the cards were divided into 6 rows and 6 columns, making spots for 36 samples on each card (Fig 1B). The samples were pipetted onto the card to cover an area of a circle with a diameter of approximately 1 cm. The volume of the applied homogenate was approximately 20 µl. The volume was approximate due to the variable nature of the homogenates, specifically their density. It was found that the best way to apply the homogenate is with a 1000 µl pipette tip, making a droplet and carefully dragging the droplet along the desired area. This way the biggest area possible can be covered while avoiding watery homogenate seeping through the downside of the card and spreading to a larger area, or conversely, piling up of a dense homogenate.

After the application of homogenates to the cards, they were left to dry at room temperature for 90 min. After drying, FTA^®^ cards with the samples can be closed and stored in a ziplock bags. To use the samples for PCR, a portion of the sample must be cut out. This can be done using a scalpel, but it is more convenient to use a hole punch. The punch or the cut-out area needs to be smaller than 1 mm^2^. Larger punches can inhibit PCR due to a high concentration of DNA. If a homogenate was particularly dense and left a crust on the surface of the card, the crust was gently scraped off with a scalpel. Based on experience, it is highly recommended to gently scrape off the surface layer as it helps to reduce the time required for the following washing steps.

**Fig 2:**
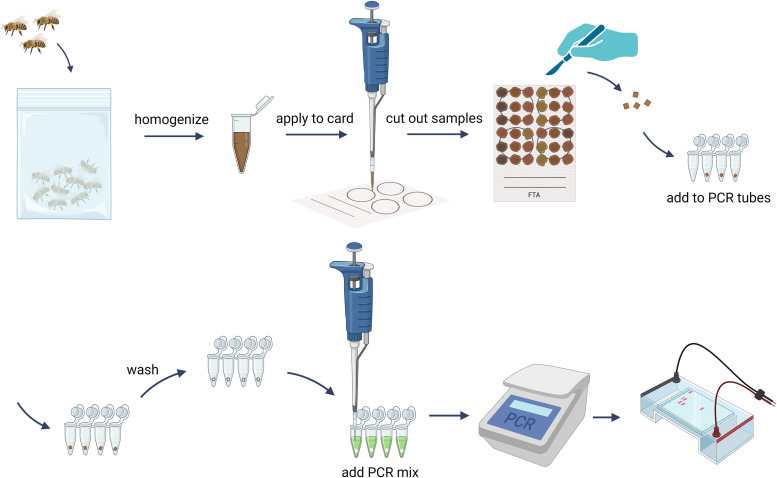
Schematic workflow of sample preparation for utilising FTA^®^ cards for PCR applications.

The card punches were transferred into PCR tubes, and 100 µl of TENT buffer (10 mM Tris-Cl, 1 mM EDTA, 12 mM NaCl, 2.5% Triton X-100, pH 8) was added. The punches were then incubated for 5 minutes. After gently shaking the tube, the punch surface should be visibly clean. After the incubation, the TENT buffer was pipetted away. The punch was washed in the same tube by adding and removing 100 µl of TENT buffer twice, followed by two washes with 100 µl of TE buffer (10 mM Tris-HCl; 0,1 mM EDTA; pH 8). This step removes residual homogenate and potential PCR inhibitors, while the DNA remains bound to the FTA^®^ card matrix. Open tubes with punches were then placed in a heat block to dry for 15 minutes at 55 °C. Alternatively, tubes can also be left to dry at room temperature for 2 hours. Dried cut-outs are then ready to be used as a template for PCR. The whole process of sample preparation with FTA^®^ cards can be seen in Fig 2.

### Polymerase chain reaction

PCR reactions were performed using DNA samples isolated by the DNeasy^**®**^ Plant Mini kit (Qiagen) (85 samples) and DNA stored on FTA^®^ cards (85 samples) to allow for comparative analysis. GoTaq DNA Polymerase (Promega, USA) was used to perform PCR according to the manufacturer’s protocol. Primers used for analysis are listed in S1 File. The amplification was initiated with a denaturation step at 95 °C for 2 min, then 34 cycles of 40 s denaturation at 95 °C, annealing at 60 °C for 30 s, and extension at 72 °C for 1 min, and ended with a final extension step at 72 °C for 5 min. The expected amplification products were tested by agarose gel electrophoresis (2.5%), stained with 2 µl of MIDORI Green Dye (Elisabeth Pharmacon, Czech Republic) and visualised in a UV transilluminator Gel Doc EZ system (BioRad, Germany). As a marker, GeneRuler 50 bp DNA ladder was used (Thermo Scientific, USA).

When a pathogen was detected, the PCR amplicons were purified using the Monarch® PCR & DNA Cleanup Kit (New England Biolabs, UK) and sent for sequencing. The nucleotide sequences obtained were compared with sequences available in the GeneBank™ database using the BLASTn tool (https://blast.ncbi.nlm.nih.gov) to confirm the identity of the pathogens. The corresponding sequence alignments can be found in S2 File. At the beginning of the study, validated positive controls were not available. Therefore, sequencing was used to confirm the specificity of the PCR assays. Once positive control materials became available, they were included in the PCR reactions. Example gel images showing the use of positive controls are provided in S2 File. As a negative control, nuclease-free water was used. A positive control for *N. apis* is not shown, as the species was only detected in one sample in the later phase of the study. However, the identity of this amplicon was confirmed by Sanger sequencing.

### Determining the limit of detection for *Nosema* spp.

The Bürker-Türk counting chamber was used for spore-counting in diluted homogenate samples. Homogenates were diluted from 1:100–1:1000 in steps of 100, and from 1:1050–1:1500 in steps of 50, resulting in a total of 20 dilution levels. The diluted homogenates were applied on FTA^®^ cards in 10 µl volumes.

### Evaluating stability of FTA^®^ cards

To evaluate the stability of samples on FTA^®^ cards, tenfold dilutions of *N. ceranae* positive samples were subjected to various forms of improper treatment to assess their impact on the integrity of the samples. The ageing of the cards was simulated by exposing them to UV light, typically used for surface sterilisation, for one hour. Additionally, the cards were subjected to prolonged heat exposure by placement in a heat block at 60 °C for three days. Oxidative damage was mimicked by storing the cards in a sealed container with cotton wool soaked in 0.3% hydrogen peroxide for five days.

### Statistics

Statistical analysis was performed in MS Excel and with the use of web calculators listed below. To compare the results of pathogen detection using FTA^®^ cards and a DNA isolating kit, Cohen’s Kappa coefficient (κ) was calculated in order to determine the degree of agreement between the two methods. To calculate κ, an online calculator available at the Idostatistics website (https://idostatistics.com/cohen-kappa-free-calculator/#risultati) was used. To interpret κ, the following ranges were used: poor agreement = less than 0.00, slight agreement = 0.00–0.20, fair agreement = 0.21–0.40, moderate agreement = 0.41–0.60, substantial agreement = 0.61–0.80, and almost perfect agreement = 0.80–1.00 [23]. Cohen’s Kappa was also used as a measure of agreement of the detection of *Nosema spp*. spores by microscopy and by PCR.

To determine the risk of occurrence of selected pathogens in autumn compared to spring and vice versa, the odds ratio (OR) was calculated using an online calculator available at the Social Science Statistics website (https://www.socscistatistics.com/biostatistics/default2.aspx). If OR=1 there was no connection between seasons and pathogen occurrence (the odds of occurrence are the same). OR<1 indicates lower odds of occurrence of infection whereas OR>1 indicates higher odds of occurrence. To compare spring and autumn samples, only apiaries with data from both seasons available were used.

Sensitivity and specificity for the detection of *N. ceranae* were calculated according to Baratloo, Hosseini [24]. Results from microscopic detection were taken as true positive. Sensitivity refers to the ability of the test to correctly identify samples that are truly positive (i.e., the proportion of true positives correctly detected), whereas specificity refers to the ability of the test to correctly identify samples that are truly negative (i.e., the proportion of true negatives correctly classified as negative).

## Results

### Microscopic detection of *Nosema spp.*

All sample homogenates were examined under a light microscope to detect *Nosema spp.* spores. *Nosema* spp. spores were microscopically detected in 36 samples (42.4%) of all tested samples (both autumn and spring). An example of a positive sample is provided in S3 File. The 49 samples (57.6%) were determined as microscopically negative.

### Application of FTA^®^ cards for sample storage and further processing for PCR

#### Possible cross-contamination of samples.

**Fig 3:**
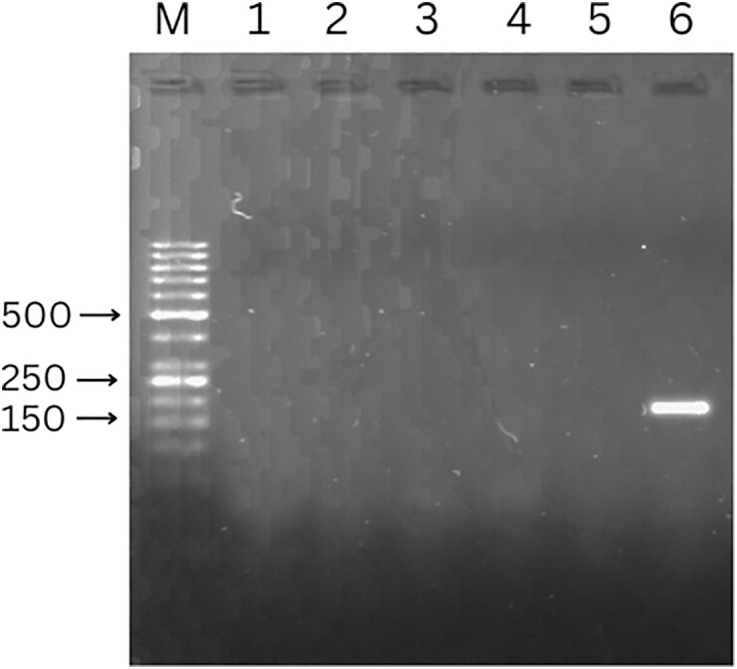
Determination of possible contamination when punching out samples from FTA^®^ cards (target: EF1-α gene, 153 bp). 1-5: repeated punches of clean parts of a sample FTA^®^ card. 6: punch from a sample area of the same FTA^®^ card (positive control). M: marker Generuler 50 bp DNA ladder.

To assess the risk of contamination during sample punching, a clean area of the card was punched immediately after punching a sample area, without cleaning the hole punch in between. This was repeated 5 times and no contamination was observed (see Fig 3).

#### Limit of detection for *N. ceranae* using FTA^®^ cards.

**Fig 4:**
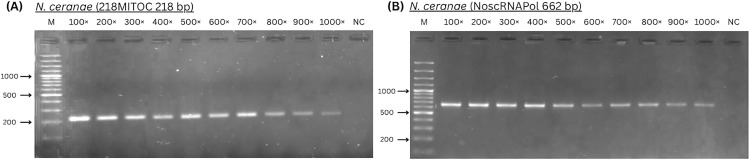
Detection of *N. ceranae* in diluted homogenates 100-1000 × . A: primer 218MITOC B: primer NoscRNAPol. M: marker Generuler 100 bp DNA ladder.

To identify the detection limit for *N. ceranae*, 2.1 × 10^6^ spores per 1 µl were counted using the Bürker-Türk counting chamber in a sample positive only for *N. ceranae*. The homogenates were diluted 100–1500× and end-point PCR using 218MITOC and NoscRNAPol primers was performed to determine the detection limit (Fig 4). The detection limit established at 1000 × dilution of homogenate, corresponding to 2.1 × 10^3^ spores per µl for both primers 218MITOC and NoscRNA Pol. Results of *N. ceranae* detection in 1050× and higher dilutions of homogenate appeared to be inconsistent when repeating the PCR with the same samples.

#### Stability of DNA on FTA^®^ cards.

Despite the applied stress conditions, i.e., UV light for 1 hour, prolonged heat (60°C for 3 days) and oxidative damage (0.3% hydrogen peroxide for 5 days), applied on the samples on FTA^®^ cards, *N. ceranae* was successfully detected across all dilutions in every experimental setup.

### Comparison of FTA^®^ cards and DNeasy^®^ Plant Mini kit for pathogen detection

#### Comparison of pathogen detection using FTA^®^ cards and DNA isolation kit across seasons.

*L. passim* was detected in 9 samples using FTA^®^ cards and 9 samples using a DNA isolation kit for autumn samples (Table 1), and in 1 sample using FTA^®^ cards and 1 sample using a DNeasy^®^ Plant Mini isolation kit for spring samples (Table 2). *N. apis* was only detected in one spring sample using FTA^®^ cards, but was not detected using the DNeasy^®^ Plant Mini kit. Moreover, it was only detected in a sample that was also positive for *N. ceranae*. *N. ceranae* was detected in 14 samples using FTA^®^ cards and only in 4 samples using the isolation kit for spring samples, and in 21 samples using FTA^®^ cards and 1 sample using the DNeasy^®^ Plant Mini kit for autumn samples. The FTA^®^ card results matched the microscopy detection results for all autumn samples, but differed in one spring sample, in which *Nosema* spores were detected using microscopy but the PCR results were negative (see sample 43 in Table 2). For *S. marcescens*, the pathogen was detected in 23 samples using FTA^®^ cards and 23 samples using the DNeasy^®^ Plant Mini kit. However, the detection results did not match in two samples (samples 5 and 40, as shown in Table 1). The comparison of selected images of agarose gels with detected products for two types of sample preparation (FTA^®^ cards vs. DNA isolation kit) is shown in S2 File.

**Table 1:** Comparison of results from microscopic detection of *Nosema spp.* and PCR detection of pathogens performed using DNeasy^®^ Plant Mini kit or with FTA^®^ cards in autumnal samples. (+) = positive infection; (−) = negative.

Autumn 2020	*L. passim*		*C. mellificae*		*S. marcescens*			*N. apis*		*N. ceranae*		*Nosema* spp. microscopy
Beekeeper number	kit	FTA	kit	FTA	kit	FTA	kit	FTA		kit	FTA	
2	−	−	−	−	−	−	−	−		−	−	−
3	−	−	−	−	−	−	−	−		−	−	−
5	−	−	−	−	+	−	−	−		−	−	−
6	−	−	−	−	−	−	−	−		−	−	−
7	+	+	−	−	+	+	−	−		−	−	−
8	−	−	−	−	−	−	−	−		−	+	+
9	−	−	−	−	−	−	−	−		−	−	−
10	−	−	−	−	−	−	−	−		+	+	+
13	−	−	−	−	+	+	−	−		−	−	−
14	−	−	−	−	−	−	−	−		−	−	−
15	−	−	−	−	+	+	−	−		−	+	+
16	−	−	−	−	+	+	−	−		−	+	+
17	−	−	−	−	−	−	−	−		−	−	−
18	−	−	−	−	−	−	−	−		−	−	−
19	−	−	−	−	+	+	−	−		−	−	−
20	−	−	−	−	−	−	−	−		−	−	−
21	−	−	−	−	−	−	−	−		−	−	−
22	−	−	−	−	+	+	−	−		−	−	−
23	−	−	−	−	−	−	−	−		−	+	+
24	−	−	−	−	−	−	−	−		−	−	−
25	+	+	−	−	+	+	−	−		−	−	−
28	−	−	−	−	+	+	−	−		−	−	−
29	−	−	−	−	+	+	−	−		−	−	−
30	−	−	−	−	−	−	−	−		−	−	−
32	+	+	−	−	−	−	−	−		−	−	−
33	−	−	−	−	+	+	−	−		−	−	−
34	−	−	−	−	−	−	−	−		−	−	−
35	−	−	−	−	−	−	−	−		−	−	−
36	−	−	−	−	+	+	−	−		−	+	+
37	−	−	−	−	+	+		−	−	−	−	−
38	−	−	−	−	+	+		−	−	−	−	−
39	−	−	−	−	+	+		−	−	+	+	+
40	−	−	−	−	−	+		−	−	−	−	−
41	+	+	−	−	+	+		−	−	+	+	+
42	−	−	−	−	−	−		−	−	+	+	+
43	−	−	−	−	+	+		−	−	−	+	+
44	−	−	−	−	+	+		−	−	−	−	−
45	+	+	−	−	−	−		−	−	−	−	−
46	+	+	−	−	+	+		−	−	−	+	+
48	+	+	−	−	−	−		−	−	−	+	+
50	−	−	−	−	+	+		−	−	−	−	−
51	−	−	−	−	−	−		−	−	−	−	−
52	−	−	−	−	−	−		−	−	−	−	−
53	−	−	−	−	+	+		−	−	−	−	−
54	−	−	−	−	+	+		−	−	−	−	−
55	−	−	−	−	−	−		−	−	−	+	+
56	+	+	−	−	−	−		−	−	−	−	−
57	−	−	−	−	+	+		−	−	−	−	−
58	−	−	−	−	−	−		−	−	−	+	+
59	+	+	−	−	−	−		−	−	−	−	−

**Table 2:** Comparison of results from microscopic detection of *Nosema spp.* and PCR detection of pathogens performed using DNeasy^®^ Plant Mini kit or with FTA^®^ cards in spring samples. (+) = positive infection; (−) = negative.

Spring 2021	*L. passim*		*C. mellificae*		*S. marcescens*		*N. apis*		*N. ceranae*		*Nosema* spp. microscopy
Beekeeper number	kit	FTA	kit	FTA	kit		kit	FTA	kit	FTA	
2	**−**	**−**	**−**	**−**	**−**	**−**	**−**	**−**	**−**	+	+
7	−	−	−	−	−	−	−	−	−	−	−
8	−	−	−	−	−	−	−	−	−	+	+
10	**−**	**−**	**−**	**−**	**−**	**−**	**−**	**−**	**−**	+	+
13	−	−	−	−	−	−	−	−	−	−	−
15	−	−	−	−	−	−	−	−	−	+	+
16	**−**	**−**	**−**	**−**	**−**	**−**	**−**	**−**	**−**	−	−
17	−	−	−	−	−	−	−	−	−	+	+
18	−	−	−	−	−	−	−	−	−	−	−
20	**−**	**−**	**−**	**−**	**−**	**−**	**−**	**−**	**−**	−	−
21	−	−	−	−	−	−	−	−	−	−	−
22	−	−	−	−	−	−	−	−	−	−	−
23	**−**	**−**	**−**	**−**	**−**	**−**	**−**	**−**	**−**	−	−
24	−	−	−	−	−	−	−	−	−	+	+
25	−	−	−	−	−	−	−	−	−	+	+
28	**−**	**−**	**−**	**−**	**−**	**−**	**−**	**−**	**−**	+	+
30	−	−	−	−	−	−	−	−	−	+	+
33	−	−	−	−	−	−	−	−	−	+	+
34	**−**	**−**	**−**	**−**	**−**	**−**	**−**	**−**	**−**	+	+
35	−	−	−	−	−	−	−	−	−	−	−
37	−	−	−	−	−	−	−	−	−	+	+
39	**−**	**−**	**−**	**−**	**−**	**−**	**−**	**−**	**−**	−	−
41	−	−	−	−	−	−	−	+	−	+	+
42	−	−	−	−	−	−	−	−	−	−	−
43	**−**	**−**	**−**	**−**	**−**	**−**	**−**	**−**	**−**	**−**	+
46	+	+	**−**	**−**	**−**	**−**	**−**	**−**	**−**	+	+
48	−	−	**−**	**−**	**−**	**−**	**−**	**−**	**−**	+	+
50	−	−	**−**	**−**	+	+	**−**	**−**	**−**	+	+
51	−	−	**−**	**−**	−	−	**−**	**−**	**−**	+	+
52	−	−	**−**	**−**	−	−	**−**	**−**	**−**	+	+
53	−	−	**−**	**−**	−	−	**−**	**−**	**−**	+	+
54	−	−	**−**	**−**	−	−	**−**	**−**	**−**	−	−
55	−	−	**−**	**−**	−	−	**−**	**−**	**−**	+	+
57	−	−	**−**	**−**	−	−	**−**	**−**	**−**	+	+
59	−	−	**−**	**−**	−	−	**−**	**−**	**−**	−	−

#### Evaluation of the measure of agreement between methods.

Cohen’s kappa coefficients were calculated to determine the measure of agreement between pathogen detection results using FTA^®^ cards, DNeasy^**®**^ Plant Mini kit and microscopy (Table 3). In the case of the detection of *L. passim* and *S. marcescens*, the agreement between results was evaluated as almost perfect in both autumn and spring samples. In spring samples, no agreement was found in the case of *N. apis* and *N. ceranae* detection. In autumn samples, the agreement of results for *N. ceranae* detection was evaluated as fair. When comparing the results from DNeasy^**®**^ Plant Mini kit and microscopy of *Nosema spp.*, the same κ values were observed as when comparing it to FTA^®^ cards results. Conversely, when comparing results for samples prepared using FTA^®^ cards with results from microscopy analysis, an almost perfect agreement was observed. Therefore, it was concluded that DNeasy^**®**^ Plant Mini kit did not provide accurate results for the detection of *Nosema* spp., whereas FTA^®^ cards did.

**Table 3:** Values of Cohen’s kappa coefficient used to determine the measure of agreement between results obtained using different methods. Hyphens mean that the pathogen was not detected, N/A mean the method could not be applied to a particular pathogen, thus kappa could not be calculated.

Pathogen	Cohen’s kappa					
	Kit vs. FTA		Kit vs. Microscopy		FTA vs. Microscopy	
	Autumn	Spring	Autumn	Spring	Autumn	Spring
*L. passim*	1.000	1.000	N/A	N/A	N/A	N/A
*C. mellificae*	–	–	N/A	N/A	N/A	N/A
*S. marcescens*	0.920	1.000	N/A	N/A	N/A	N/A
*N. apis*	–	0.000	N/A	N/A	N/A	N/A
*N. ceranae*	0.365	0.000	N/A	N/A	N/A	N/A
*Nosema spp.*	N/A	N/A	0.365	0.000	1.000	0.940

#### Sensitivity and specificity of used methodology.

The sensitivity and specificity of PCR using FTA^®^ cards or DNeasy^®^ Plant Mini Kit for detecting *N. ceranae* were assessed, with both methods used without prior spore lysis. FTA^®^ cards demonstrated exceptional performance, achieving a sensitivity of 97.2%, reflecting their ability to accurately identify nearly all microscopy-positive cases, and a specificity of 100%, indicating no false positives. In contrast, the DNeasy^®^ Plant Mini Kit exhibited markedly lower sensitivity at 11.8%, likely due to the absence of a spore lysis step, while maintaining a specificity of 100%. These results underscore the efficacy of FTA^®^ cards for reliable detection of *N. ceranae*.

### Pathogen prevalence in autumn and spring samples

Pathogen prevalence was calculated as the percentage of positive samples among all tested samples in autumn 2020 and spring 2021, respectively. For the calculation, only results obtained using FTA^®^ cards were used. The most prevalent pathogen in autumn samples was *S. marcescens* (46%) followed by *N. ceranae* (28%) and *L. passim* (18%). No *C. mellificae* and *N. apis* was detected within autumn samples. Within spring samples, *N. ceranae* dominated with 60% prevalence; other pathogens were detected only in one sample (*N. apis*, *S. marcescens*, *L. passim*) or not at all (*C. mellificae*). The results are listed in Table 4.

**Table 4:** Comparison of pathogen prevalence in honey bee samples from autumn 2020 and spring 2021. The listed prevalences were calculated with data obtained by using FTA^®^ cards.

Pathogen	Autumn 2020 (total 50 samples)		Spring 2021 (total 35 samples)	
	positive samples	prevalence	positive samples	prevalence
*L. passim*	9	18%	1	2.8%
*C. mellificae*	0	0%	0	0%
*S. marcescens*	23	46%	1	2.8%
*N. apis*	0	0%	1	2.8%
*N. ceranae*	14	28%	21	60%

### Agreement between pathogen occurrence in autumn 2020 and spring 2021 and the odds of infection

To determine whether there was an agreement between the occurrence of the same pathogens in autumn 2020 and in spring 2021, κ coefficients were calculated using data obtained from FTA^®^ cards (see Table 5). In the case of *S. marcescens*, slight agreement was observed. For *L. passim*, fair agreement was observed, whereas poor agreement was found in the case of *N. apis* and *N. ceranae*.

**Table 5:** Evaluation of agreement between pathogen occurrence in the same colonies in autumn 2020 and spring 2021.

Pathogen	autumn 2020 *vs.* spring 2021
	Cohen’s kappa
*L. passim*	0.250
*C. mellificae*	–
*S. marcescens*	0.060
*N. apis*	0.000
*N. ceranae*	−0.022

To evaluate the odds of occurrence of infection in colonies in autumn 2020 compared to spring 2021 and *vice versa*, OR values were calculated (Table 6). *L. passim* was more prevalent in colonies in autumn 2020 compared to the situation in the same colonies in spring 2021. A similar result was observed in the case of *S. marcescens*, where the odds of occurrence of infection were significantly higher in autumn 2020. Conversely, for *N. ceranae*, higher odds of occurrence were observed in spring 2021 compared to autumn 2020. Although the sample size for some pathogen detections, especially in spring, was very low, Odds Ratios were calculated to illustrate the direction of seasonal variation. These values should be interpreted with caution and are not intended to support statistically robust conclusions.

**Table 6:** Comparison of the odds of occurrence of pathogens in bee colonies to infection in autumn 2020 and in spring 2021, determined by odds ratio. To compare autumn 2020 results to spring 2021, the denomination OR1 was used, and to compare spring 2021 to autumn 2020, OR2 was used.

Pathogen	Autumn 2020/Spring 2021	Spring 2021/Autumn 2020
	OR1	OR2
*L. passim*	7.03	0.14
*C. mellificae*	–	–
*S. marcescens*	32.11	0.03
*N. apis*	–	–
*N. ceranae*	0.35	2.88

### Co-infection of pathogens

In autumn, 32% of colonies were not infected with any of the selected pathogens, whereas in spring it was 40% of colonies. 48% of colonies in autumn were infected with one pathogen, 16% with two different pathogens and 4% with three different pathogens. No colony in autumn was infected with more than three pathogens. In spring, more than half of the colonies (51%) were infected with one pathogen. The remaining colonies in spring (9%) were infected with two kinds of pathogens.

### Geographical distribution of pathogens within the Czech Republic

The selected pathogens were detected in apiaries across various regions of the Czech Republic. The geographical distribution of the detected pathogens in Autumn 2020 and Spring 2021 is presented in –. *C. mellificae* was not included in the maps due to the absence of positive detection in analysed apiaries. In autumn 2020, *L. passim* appeared in multiple regions, with a relatively higher number of positive detections in the central, north-western, and eastern parts of the country, while no positive cases were observed in the southern regions. This may suggest a spatial clustering in certain areas during that time. In Spring 2021, however, *L. passim* was detected in only a single location, and most previously positive apiaries tested negative. In autumn, *S. marcescens* was detected at multiple locations, showing a largely random distribution across most regions of the country; however, relatively few positive detections were observed in the eastern part of Czechia. In spring, only one location tested positive, while most sites that had previously shown presence of *S. marcescens* in autumn were negative. There were no positive *N. apis* detections in autumn 2020. In spring 2021, a single positive location was recorded near the center of the country. Notably, this site also tested positive for *N. ceranae* in both autumn and spring samples.

**Fig 5:**
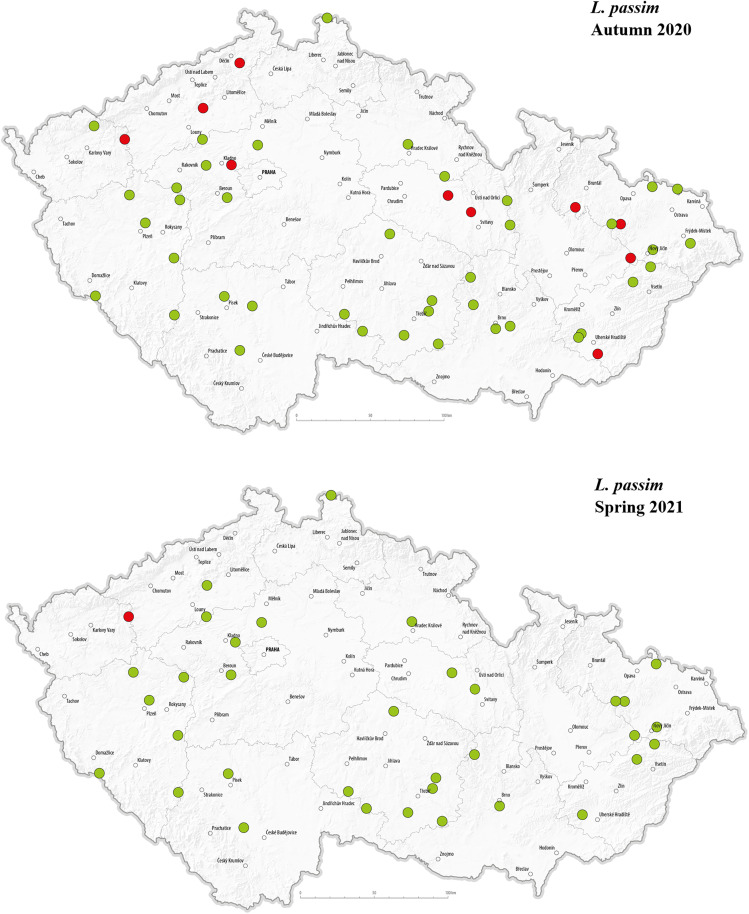
Geographical distribution of tested hives with a representation of positive (red dots) and negative (green dots) detection of *L. passim* in the Czech Republic in autumn 2020 and spring 2021.

**Fig 6:**
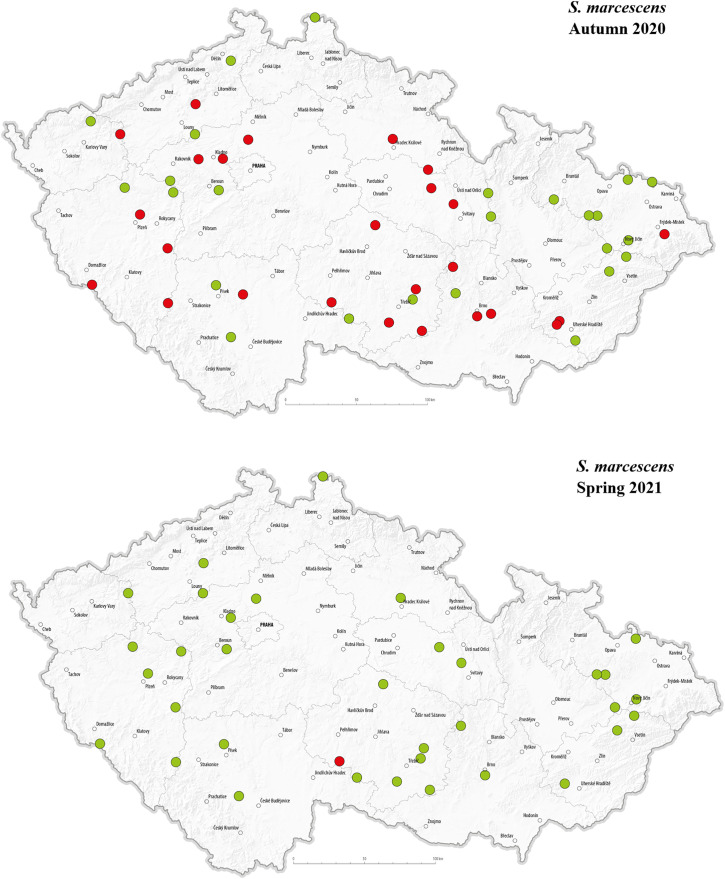
Geographical distribution of tested hives with a representation of positive (red dots) and negative (green dots) detection of *S. marcescens* in the Czech Republic in autumn 2020 and spring 2021.

**Fig 7:**
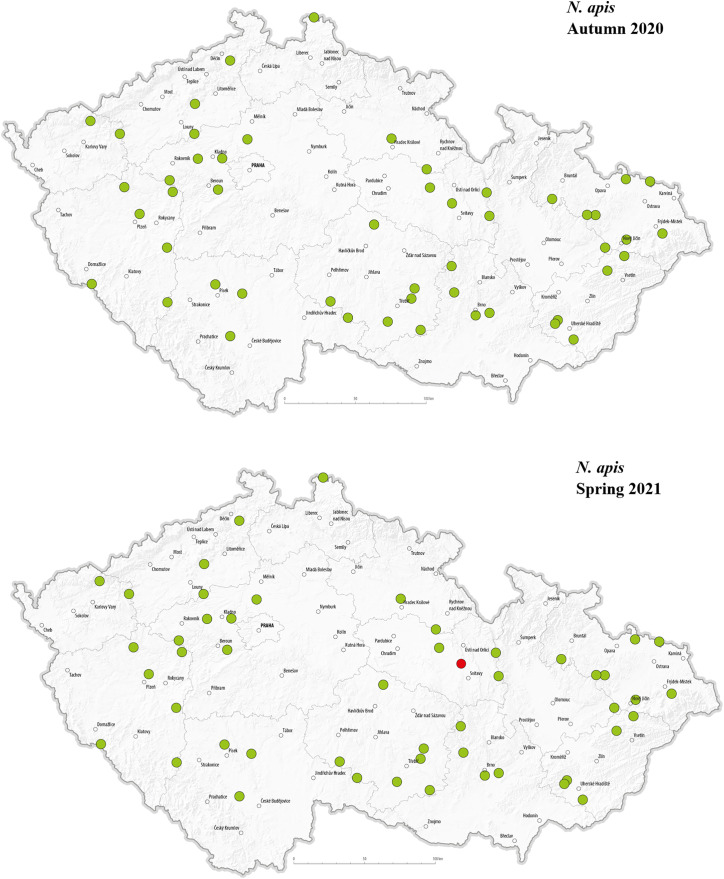
Geographical distribution of tested hives with a representation of positive (red dots) and negative (green dots) detection of *N. apis* in the Czech Republic in autumn 2020 and spring 2021.

**Fig 8:**
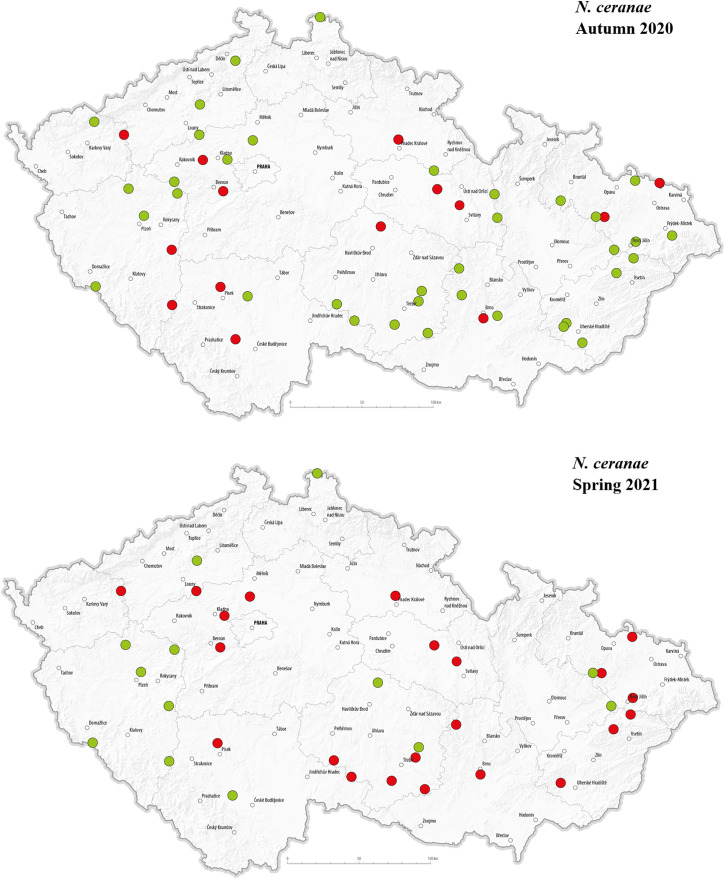
Geographical distribution of tested hives with a representation of positive (red dots) and negative (green dots) detection of *N. ceranae* in the Czech Republic in autumn 2020 and spring 2021.

In autumn 2020, *N. ceranae* detections appeared relatively localised, with more positive sites observed in the eastern and north-eastern parts of the country compared to central and southern regions. In spring 2021, positive detections were recorded in a greater number of locations, covering most of the country except the south-west. Although the sampling was not systematic, the data suggest a broader distribution of *N. ceranae* in spring compared to autumn.

## Discussion

FTA^®^ cards are utilised for diverse applications across various fields. They are routinely used in clinical laboratories for blood sample storage and can also be employed for storing and transporting viral RNA from sites where it is difficult to ensure optimal conditions for conventional sampling methods [25]. They have also been used for pathogen detection in entomology, for instance, for the detection of circulating viruses in mosquitoes [26] or *Nosema bombycis* in silkworm eggs [27]. To our knowledge, this is the first study to use FTA^®^ cards for pathogen detection in honey bees, with successful application to field samples. For use with plant samples, such as leaves, it is possible to apply the samples by only pressing them onto the card [28]. Nevertheless, this approach is not ideal for honey bees, as observed during protocol development. When pressing a bee onto the card, it is impossible to get an even distribution of samples, particularly gut content, which is critical for detecting intestinal pathogens. Additionally, this method is inefficient in terms of FTA^®^ card space utilisation. Honey bee homogenates were used instead to address these challenges. The use of extraction bags facilitated the rapid homogenisation of a larger number of bees, i.e., 20 in this study. This approach allowed 36 samples of honey bee homogenates to be applied to a single FTA^®^ card, with at least 20 card punches obtained from each sample. This was more than sufficient for the purposes of this study and is also suitable for field screening of multiple pathogens.

The cost of a single FTA^®^ card is approximately 7 USD (a 100-card bundle of QIAcard^®^ FTA^®^ Classic costs 692 USD on Qiagen’s website in 2025), allowing for DNA isolation from 36 samples at approximately 0.2 USD per sample, excluding additional laboratory materials. This is significantly more affordable than the DNeasy® Plant Mini kit, which costs around 200 USD for 36 samples (5.6 USD per sample, with a 250 sample-preparation kit priced at 1380 USD). There are notable differences in the time requirements and complexity of each method. The primary advantage of using the kit is that DNA isolation is performed only once. Afterwards, the stored frozen DNA simply needs to be thawed and can be immediately used for PCR reactions. In contrast, FTA^®^ cards require additional time for sample preparation before each use. However, the preparation of the PCR reaction mixture is faster with FTA^®^ cards, as the premix is directly applied onto the card in a PCR tube or plate, eliminating the need to pipette the DNA separately. This may also minimise the risk of cross-contamination since the DNA is immobilised on the card, while using kit-isolated DNA may cause contamination by aerosolised DNA [29,30]. The DNA isolation process itself is easier with the use of FTA^®^ cards than with the column-based DNeasy^®^ Plant Mini kit. However, the time needed for both methods is comparable. Each method offers unique time advantages and disadvantages, depending on the specific steps of the protocol. It should also be noted that, unlike DNA in solution, DNA on an FTA^®^ card cannot be used for quantitative PCR unless eluted, as the DNA concentration on individual card punches is unknown.

When detecting pathogens using FTA^®^ cards and DNeasy^®^ Plant Mini kit, it was observed that the results matched for 3 out of 5 pathogens that were being detected. For the detection of *Nosema spp.,* FTA^®^ cards performed significantly better than DNeasy^®^ Plant Mini kit. Out of 36 *Nosema*-positive samples determined based on microscopy results, 35 samples were correctly identified using FTA^®^ cards, whereas only 4 were correctly identified using the kit. *Nosema* spp. spores are likely too resilient, and the lysis step used was insufficient to release their DNA using the DNeasy^®^ Plant Mini kit. As suggested by Fries, Chauzat [13], the isolation of *Nosema spp.* DNA using DNeasy^®^ Plant Mini kit requires prior centrifuging of the homogenate and freezing and pulverising the pellet multiple times to successfully break open *Nosema* spore walls. This is a lengthy process unnecessary for other selected pathogens. The sensitivity of *N. ceranae* detection using FTA^®^ cards was calculated to be nearly 100%. This highlights FTA^®^ cards as a promising and reliable alternative to traditional DNA isolation kits, providing highly accurate results for pathogen detection in honey bee homogenates. Furthermore, for *Nosema* spp., FTA^®^ cards proved to be more time-efficient, as the additional steps described by Fries, Chauzat [13] required for DNA isolation of these microsporidia using the kit significantly increased the sample preparation time.

The detection limit for the detection of *N. ceranae* using FTA^®^ cards was determined to be 2168 spores. This is comparable with the detection limit of 2877 spores reported by Erler, Lommatzsch [31] using the DNeasy^®^ Plant Mini kit with prior spore lysing and the same primers used in this study. The limit of detection for other selected pathogens could not be determined, as they could not be detected using microscopy.

Rahikainen, Palo [21] highlight the long-term stability of DNA stored on FTA^®^ cards, with samples maintaining sufficient quality for genetic analyses even after 16 years of storage. This stability is attributed to the chemically treated matrix of the FTA^®^ cards, which protects DNA from nucleases, oxidative agents, and bacterial degradation. To address the stability of samples on FTA^®^ cards in this study, positive samples stored on FTA^®^ cards have been used as positive controls for over two years, and they remain fully effective for PCR analysis, demonstrating their stability. These cards are stored at room temperature in sealed plastic bags, which protects them from dust and other environmental factors. The stability of DNA on FTA^®^ cards without the need for refrigeration greatly facilitates the long-term storage of positive controls, which is particularly practical for laboratories with limited space in freezers.

The DNA on FTA^®^ cards were found to remain stable even under challenging conditions, including exposure to high temperatures, UV radiation, and oxidative damage. This capability makes FTA^®^ cards an ideal tool for preserving the integrity of DNA samples. One of the main advantages of the protocol developed in this study is its adaptability for direct use by beekeepers, bee inspectors or technicians. Because the method does not require cold storage or advanced laboratory equipment, we propose it could be simplified into a practical field kit. Beekeepers could be provided with a small sampling set containing: a sterile microcentrifuge tube and disposable plastic pestle, nuclease-free water, pre-cut FTA^®^ card strips, and a sterile zip-lock bag. With minimal instructions, beekeepers could homogenize a few adult bees in the tube, apply a drop of the homogenate onto the card, let it air-dry, and then send the sample by post to the laboratory. This simple, low-cost approach would reduce the risk of DNA degradation during transport and enable more widespread, decentralised participation in pathogen monitoring. By empowering beekeepers to contribute samples directly, this protocol has the potential to significantly improve the scope and resolution of honey bee health surveillance.

FTA^®^ cards together with end-point PCR were utilised to detect bee pathogens and determine their prevalence in autumn 2020 and spring 2021 in various regions of the Czech Republic. Our results coincide with those of previously published studies. Emsen, De la Mora [32] report that in North America, the highest rates of *N. ceranae* infection, its prevalence and spore lifespan, were recorded in spring and summer. The lowest were observed in autumn. In Germany, Gisder, Schüler [33] also noted an increase in the prevalence of *N. ceranae* during the summer, which they attribute to a higher proliferation potential of *N. ceranae* in the warmer months. *N. apis* was detected in only one of the investigated bee colonies, which confirms the already documented displacement of *N. apis* by the more virulent species *N. ceranae* [34,35]. This was also reported in the Czech Republic by Kamler, Titěra [36]. At the same time, *N. apis* was detected in a sample that was also positive for *N. ceranae*. This co-infection is not very common [33].

*Serratia* species can sometimes be found naturally in low abundance in the digestive tract of bees as part of their microbiome [37]. However, the presence of *S. marcescens* in higher abundance is considered a signifier of an atypical microbial composition [38]. In this study, it was exclusively detected in autumn 2020 in almost half of the colonies (46%). This difference may be due to possible differences in the nutrition of spring 2021 and autumn 2020 bee colonies, as nutrition can affect the diversity of the microbiome [39,40]. Kešnerová, Emery [41] also report that bees may have mechanisms that increase resistance to opportunistic pathogens in the winter months, which could explain why bees after winter in spring 2021 were less infected with this pathogen compared to autumn 2020 of the previous year.

*C. mellificae* was not detected in any bee colony tested. This may confirm the hypothesis that the previously frequently detected *C. mellificae* was, in fact, its relative *L. passim* [7], which, in this study, was detected in almost a quarter of bee colonies in autumn 2020. These results coincide, for example, with those in Japan in 2018 and 2019, where the prevalence of *C. mellificae* was 0% and for *L. passim* ranged from approximately 16–70% in different regions [42]. *L. passim* is also reported to be more abundant in the colder months [43]. In the Czech Republic in 2021, *C. mellificae* was reported by Mráz, Hýbl [44] as one of the most prevalent pathogens alongside *N. ceranae* and *L. passim*, while *N. apis* was absent from all colonies studied. Although they used the same primers as in our study, their methodology differed by employing an alternative DNA isolation method and a different polymerase. Vočadlová [45] also reported *L. passim* was found in 71% of samples collected in the western part of Czechia, whereas *C. mellificae* was not detected in any sample.

Colonies that tested positive for specific pathogens in autumn did not consistently show infections with the same pathogens in spring. This lack of continuity suggests that the presence of these pathogens may be transient or influenced by seasonal factors, environmental changes, or colony health dynamics over winter. These findings suggest that infections detected in autumn do not reliably predict pathogen presence in the subsequent spring, highlighting the complex, potentially fluctuating nature of pathogen persistence within bee colonies across seasons. While similar seasonal trends have been observed in other regions, this represents one of the first such observations in the Czech Republic.

The collected data also provide an initial view of pathogen distribution across the Czech Republic in autumn and following spring. However, without previous research mapping pathogen distribution in the Czech Republic, we cannot directly compare these patterns with other studies.

While the findings offer useful insight into pathogen prevalence and demonstrate the applicability of FTA® cards, several limitations must be acknowledged. The bee samples were provided voluntarily by beekeepers and were not collected through a structured, randomised sampling scheme. In some cases, suboptimal transport or storage conditions led to the exclusion of spring samples. Moreover, the number of positive detections for certain pathogens was low, limiting statistical power and restricting the ability to perform formal statistical comparisons at the country level. Nevertheless, our sampling approach follows principles described by van Engelsdorp, Eugene [46] in the COLOSS BEEBOOK, where voluntary and observational data collection is considered a valid strategy for epidemiological screening and method development. Thus, although our dataset is not representative at the national level, it offers a relevant exploratory view of pathogen presence in field conditions and supports the potential of the proposed method for future monitoring programs.

Although this study focused on DNA-based detection of intestinal pathogens, FTA^®^ cards also hold promise for RNA preservation and virus diagnostics. Previous studies have demonstrated that RNA can be successfully recovered from FTA cards using suitable elution buffers, followed by DNase treatment and RT-PCR. This approach has already been used in virology and entomological research [25]. While we did not test RT-PCR for honey bee RNA viruses in this study, there is no technical barrier to applying the method for such targets. However, reverse transcription directly on card punches may be less efficient due to possible inhibition by card reagents or residual DNA, making an intermediate RNA elution step advisable. Testing the utility of FTA^®^ cards for detecting RNA viruses represents an important direction for future research and could expand the applicability of this sampling method to include both DNA and RNA pathogens.

## Conclusions

This study demonstrates the effectiveness of FTA^®^ cards as a reliable, cost-effective, and practical alternative to DNA-isolation kits for pathogen detection in honey bee homogenates. The cards preserved DNA integrity even under improper storage conditions, such as high temperatures, UV exposure, and oxidative damage, making them well-suited for field sampling and transport. Beekeepers can utilise FTA^®^ cards to simplify sample collection and prevent degradation, ensuring accurate diagnostics without the need for refrigeration. For *N. ceranae*, FTA^®^ cards outperformed traditional DNA isolation kits in both sensitivity and time efficiency, eliminating the need for labour-intensive spore lysis protocols. The results also provide insights into seasonal variations in pathogen prevalence and underscore the complex dynamics of infections within bee colonies. Overall, this study highlights the potential of FTA^®^ cards to advance pathogen monitoring.

## Supporting information

S1 File Primers used for endpoint PCR detection of selected pathogens. (DOCX)

S2 File Sequence alignments and agarose gels after end point PCR. (DOCX)

S3 File Microscopic confirmation of *Nosema* spores. (TIFF)

S4 File Original figures of agarose gels which were imaged using the Bio-Rad Gel Doc EZ Documentation System and analyzed with Image Lab Software (Bio-Rad Laboratories). (PDF)
