# Supplementary material for: Efficient pathogen screening in honey bees: Application of FTA® cards for DNA storage and PCR analysis
Source: PLoS One. 2025 Oct 30;20(10):e0334066. doi: 10.1371/journal.pone.0334066 (PMC12574871; doi:10.1371/journal.pone.0334066)
Supplement: S1 File — (DOCX) [file pone.0334066.s001.docx]

Table S1: Primers used for endpoint PCR detection of selected pathogens.

| **gene/**  **pathogen** | **Primer name** | **Reference** | **Primer 5´→ 3´** | | **amplicon size (bp)** |
| --- | --- | --- | --- | --- | --- |
|  |  |  | **Forward** | **Reverse** |  |
| *Honey bee* (*EF-1 alpha*) |  | (Lourenço et al., 2008) | GGAGATGCTGCCATCGTTAT | CAGCAGCGTCCTTGAAAGTT | 153 |
| *S. marcescens* |  | (Bussalleu & Althouse, 2018) | GGTGAGCTTAATACGTTCATCAA | AATTCCGATTAACGCTTGCAC | 107 |
| *N. apis*  *N. ceranae* | NosaRNAPol  +  NoscRNAPol  (duplex PCR) | (Gisder & Genersch, 2013) | AGCAAGAGACGTTTCTGGTACCTCA | CCTTCACGACCACCCATGGCA | 297 |
|  |  |  | TGGGTTCCCTAAACCTGGTGGTTT | TCACATGACCTGGTGCTCCTTCT | 662 |
| *N. ceranae* | 218MITOC | (Martin-Hernandez et al., 2007) | CGGCGACGATGTGATATGAAAATATTAA | CCCGGTCATTCTCAAACAAAAAACCG | 218-219 |
| *C. mellificae* | CmCytb | (Stevanovic et al., 2016) | AGTTTGAGCTGTTGGATTTGTT | AACCTATTACAGGCACAGTTGC | 140 |
| *L. passim* | LpCytb | (Stevanovic et al., 2016) | CGAAGTGCACATATATGCTTTAC | GCCAAACACCAATAACTGGTACT | 247 |
