# Supplementary material for: Efficient pathogen screening in honey bees: Application of FTA® cards for DNA storage and PCR analysis
Source: PLoS One. 2025 Oct 30;20(10):e0334066. doi: 10.1371/journal.pone.0334066 (PMC12574871; doi:10.1371/journal.pone.0334066)
Supplement: S2 File — (DOCX) [file pone.0334066.s002.docx]

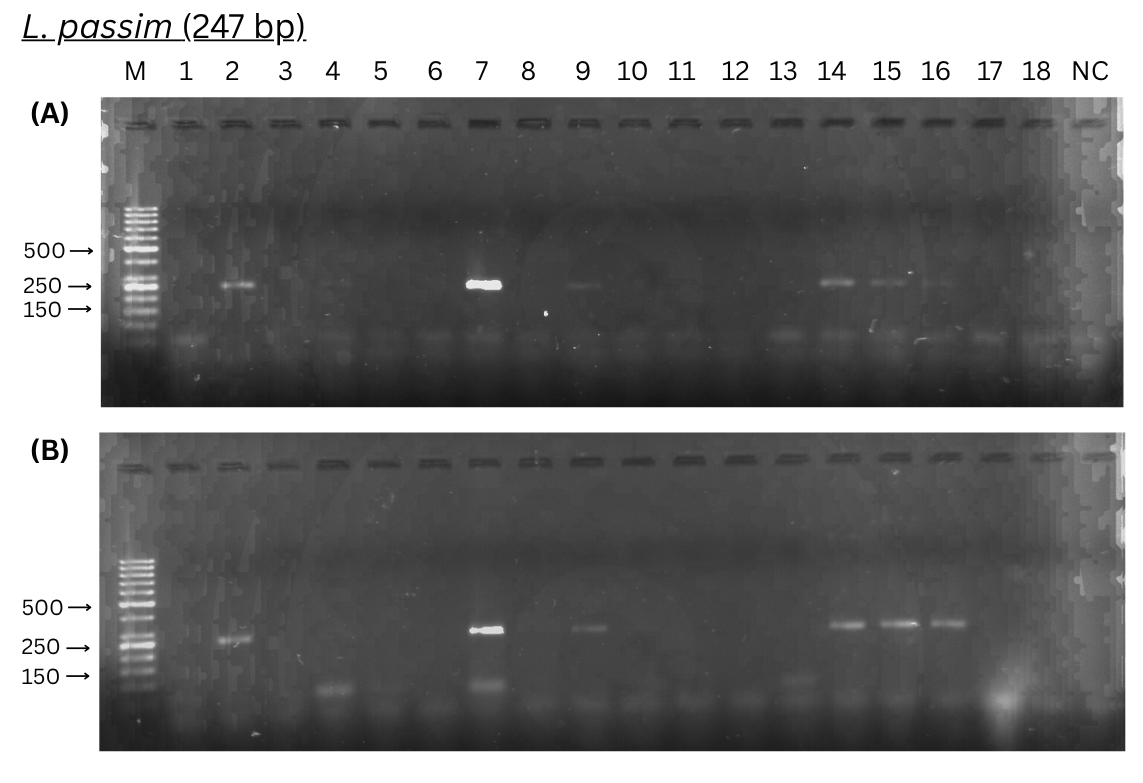


Figure S2-1 Comparison of endpoint PCR results for the detection of L. passim. (A) DNeasy® Plant Mini kit results. (B) FTA cards results. M: marker Generuler 50 bp; 1-18: selected samples of homogenates, NC: negative control.


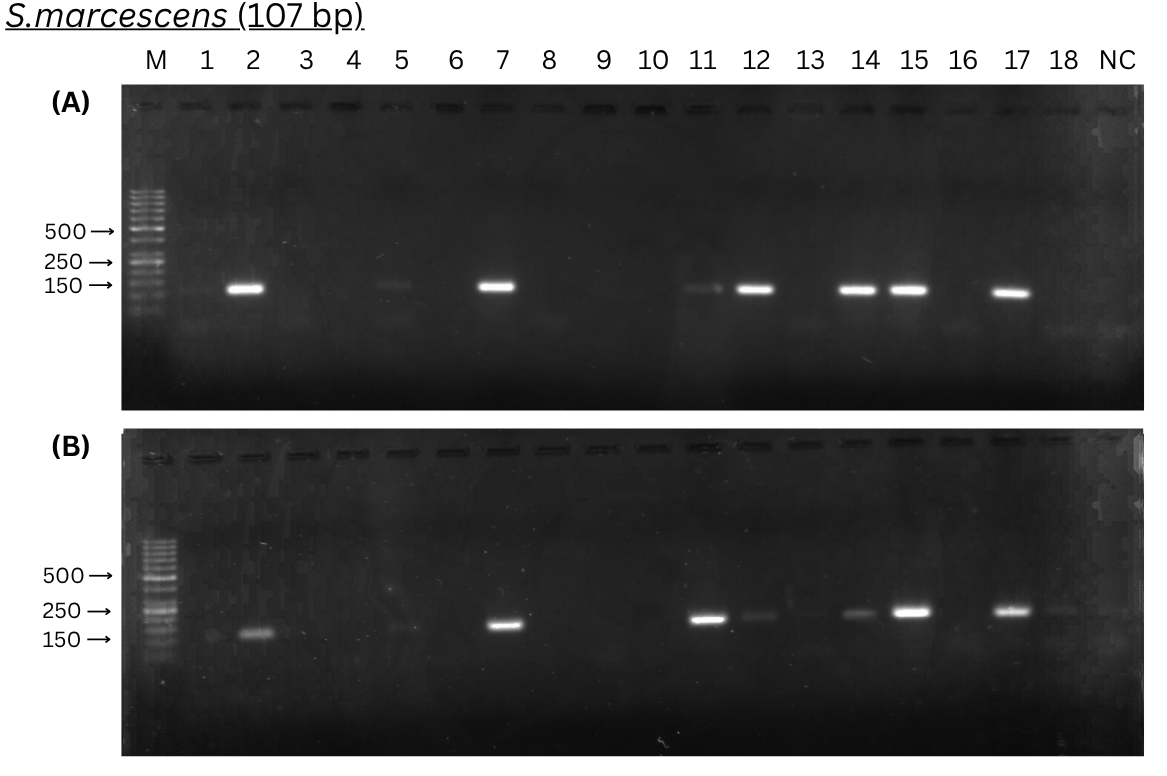


Figure S2-2 Comparison of endpoint PCR results for the detection of S. marcescens. (A) DNeasy**^®^** Plant Mini kit results. (B) FTA cards results. M: marker Generuler 50 bp; 1-18: selected samples of homogenates, NC: negative control.


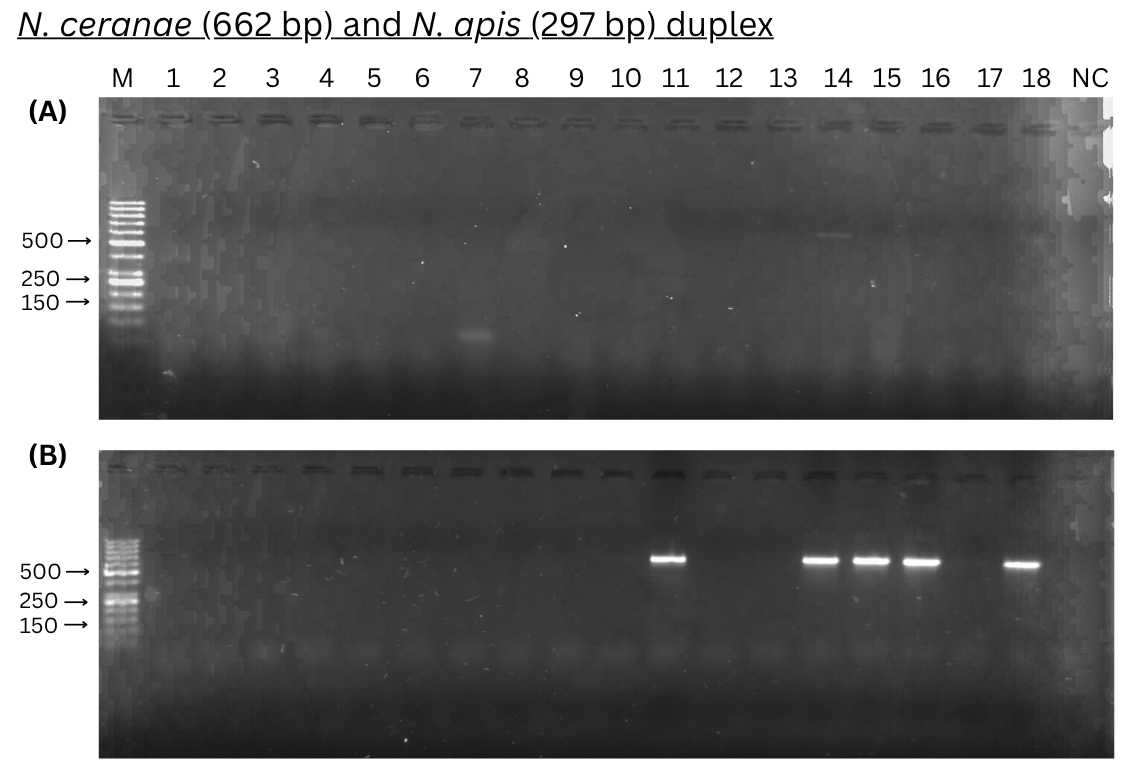


Figure S2-3 Comparison of endpoint PCR results for the detection of N. apis and N. ceranae. (A) DNeasy® Plant Mini kit results. (B) FTA cards results. M: marker Generuler 50 bp; 1-18: selected samples of homogenates, NC: negative control.


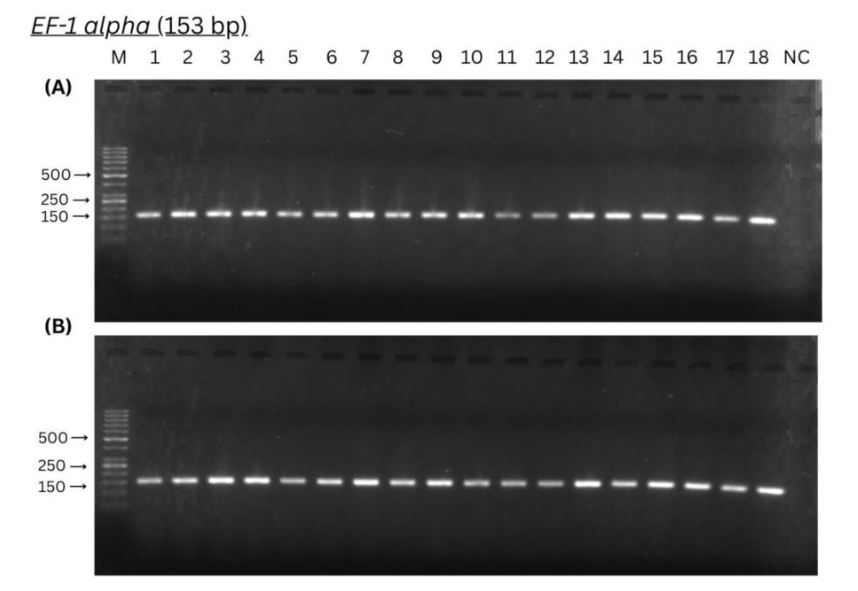


Figure S2-4 Comparison of endpoint PCR results for the housekeeping gene EF1-alpha. (A) DNeasy® Plant Mini kit results. (B) FTA cards results. M: marker Generuler 50 bp; 1-18: selected samples of homogenates, NC: negative control.


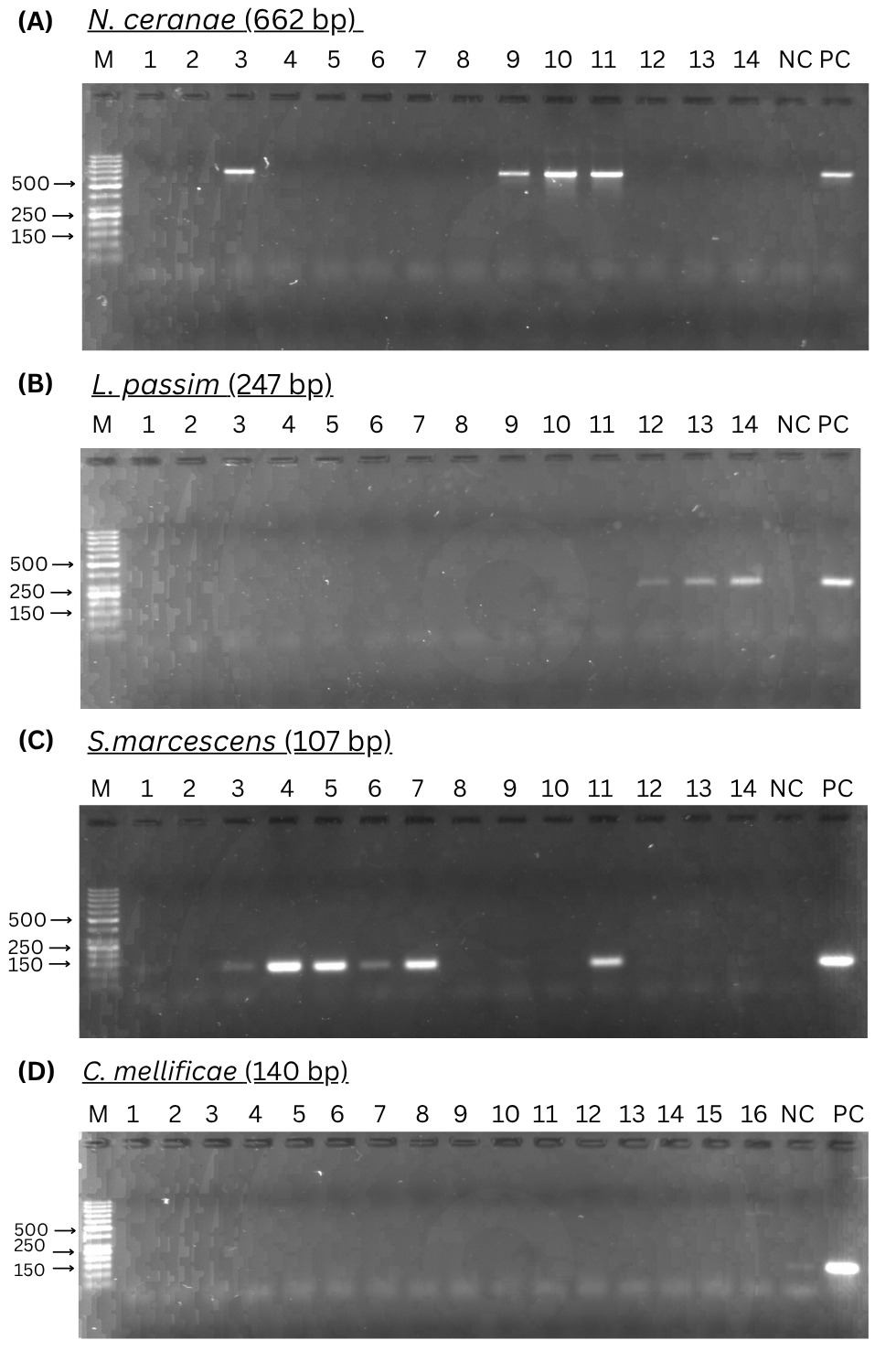


Figure S2-5 Representative gel images showing positive controls for selected pathogens excluding N. apis. M: marker Generuler 50 bp; 1-16: selected samples of homogenates; NC: negative control; PC: positive control.


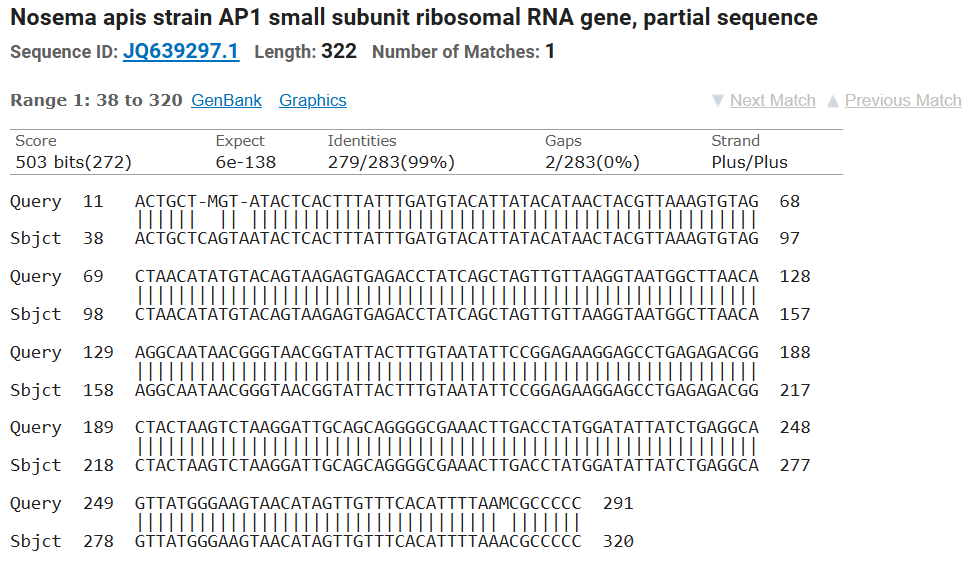


Figure S2-6 BLASTn alignment confirming the identity of the N. apis PCR amplicon.


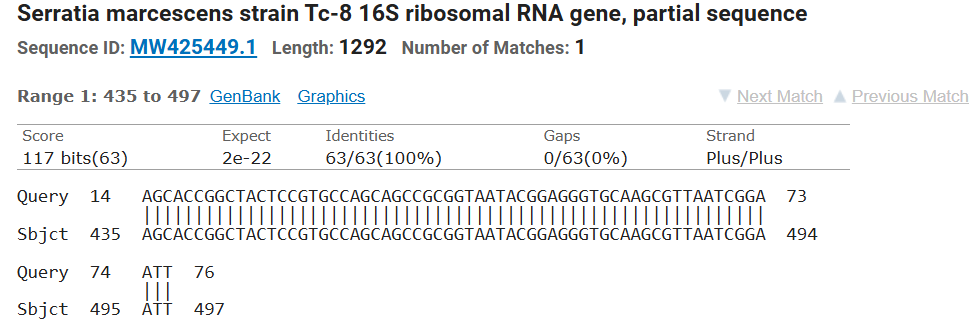


Figure S2-7 BLASTn alignment confirming the identity of the S. marcescens PCR amplicon.


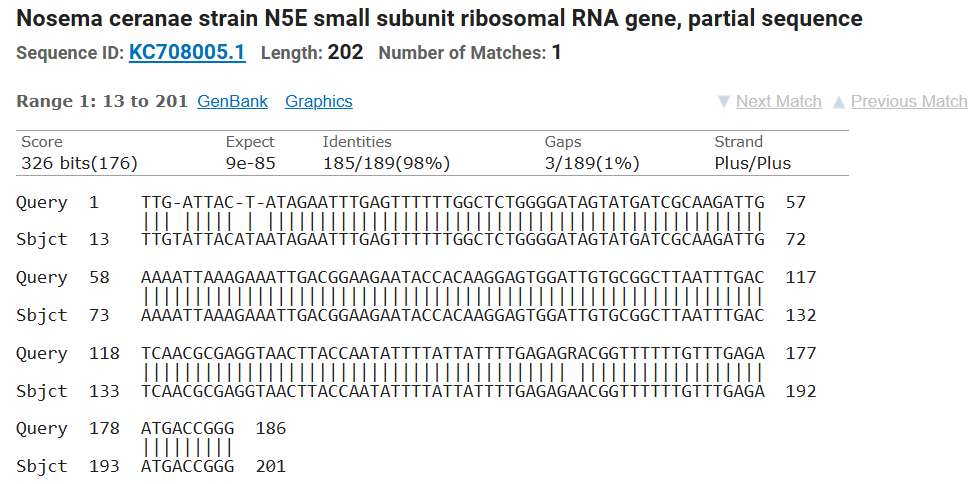


Figure S2-8 BLASTn alignment confirming the identity of the N. ceranae PCR amplicon.


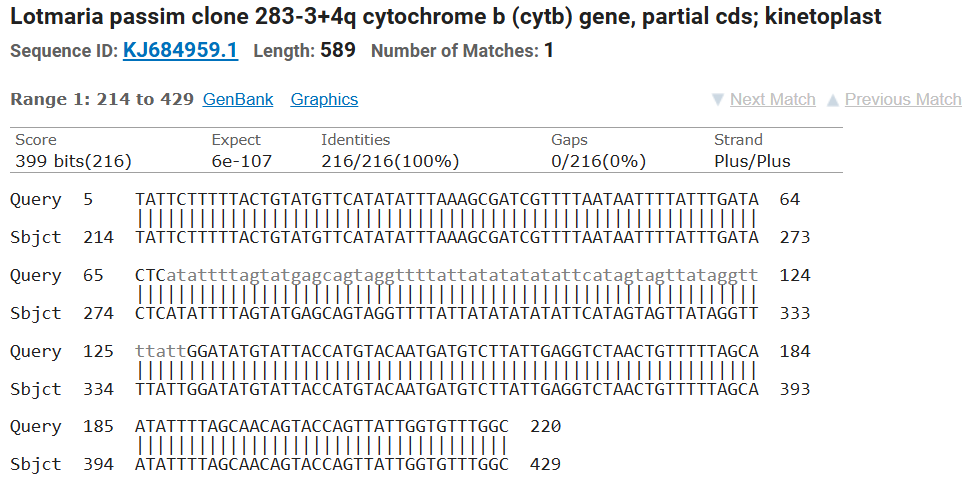
Figure S2-9 BLASTn alignment confirming the identity of the *L. passim* PCR amplicon.
