# Supplementary figures and images for: Efficient pathogen screening in honey bees: Application of FTA® cards for DNA storage and PCR analysis
Source: PLoS One. 2025 Oct 30;20(10):e0334066. doi: 10.1371/journal.pone.0334066 (PMC12574871; doi:10.1371/journal.pone.0334066)

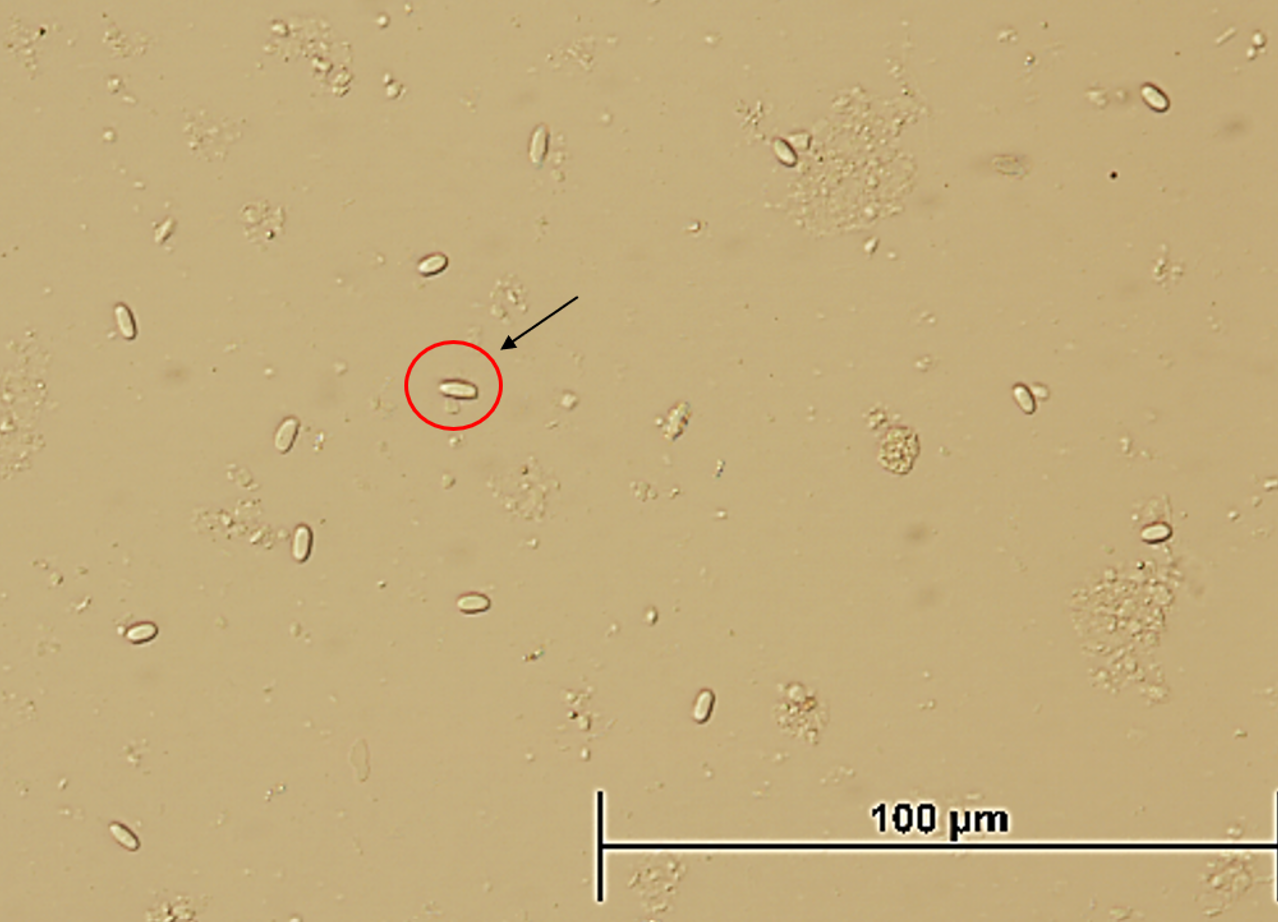

Supplement: S3 File — (TIFF) [file pone.0334066.s003.tiff]
