# Supplementary material for: Efficient pathogen screening in honey bees: Application of FTA® cards for DNA storage and PCR analysis
Source: PLoS One. 2025 Oct 30;20(10):e0334066. doi: 10.1371/journal.pone.0334066 (PMC12574871; doi:10.1371/journal.pone.0334066)

All figures in this document represent agarose gels which were imaged using the Bio-Rad Gel Doc EZ Documentation System and analyzed with Image Lab Software (Bio-Rad Laboratories)

Figure 3

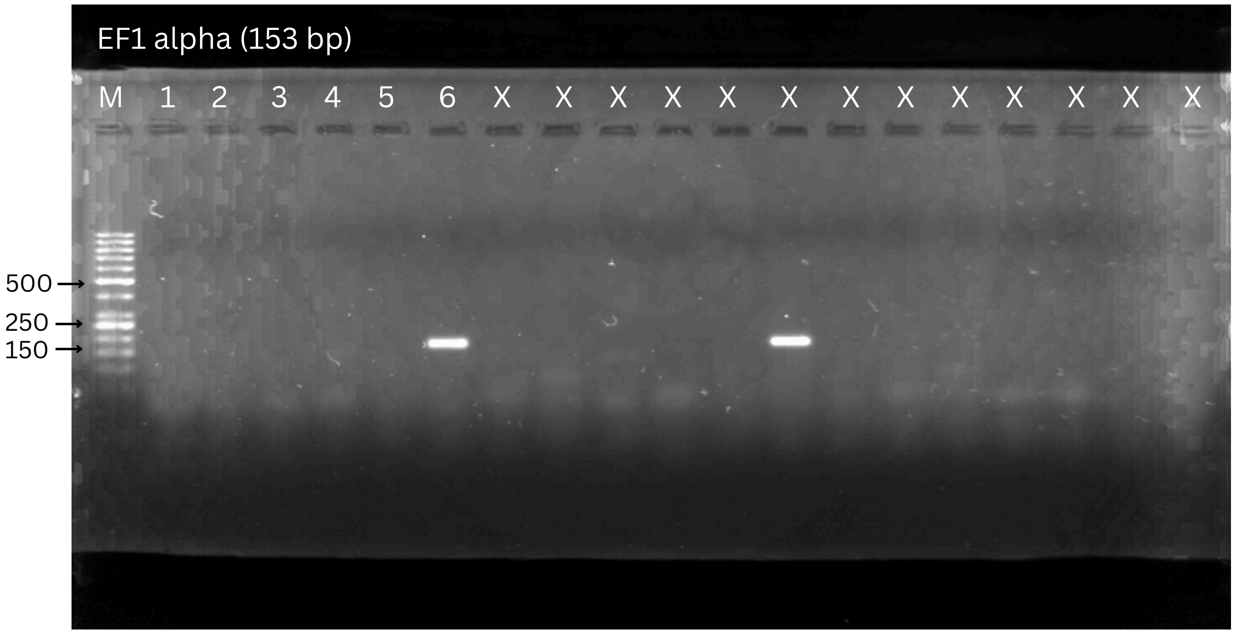

Figure 4A

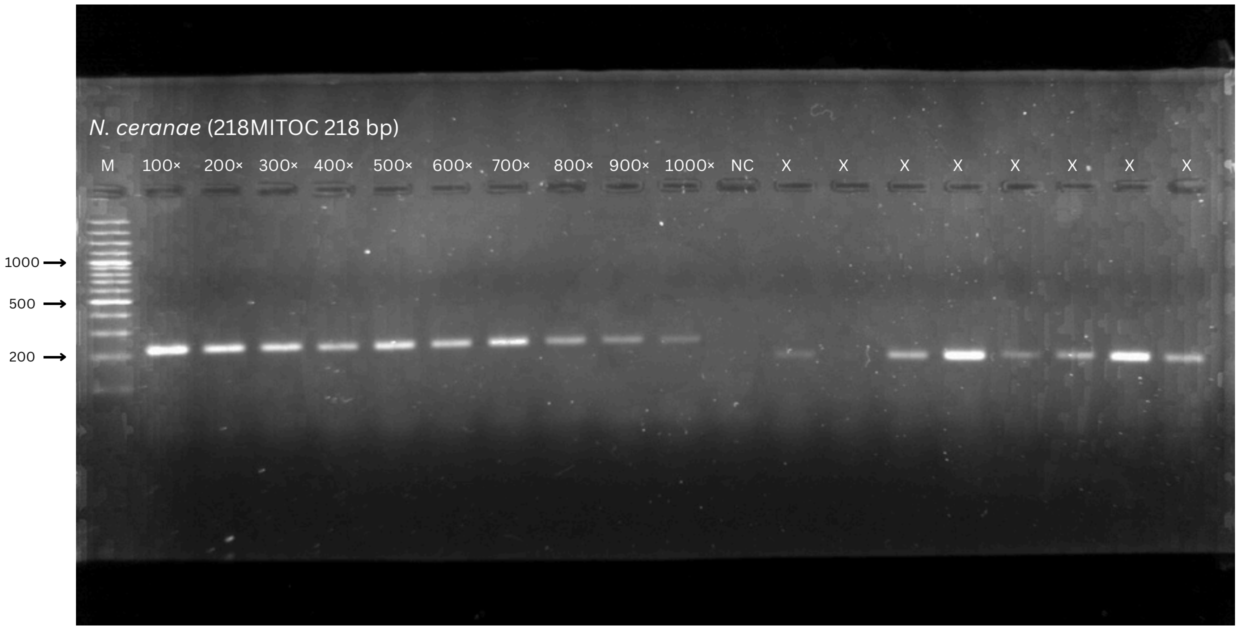

Figure 4B

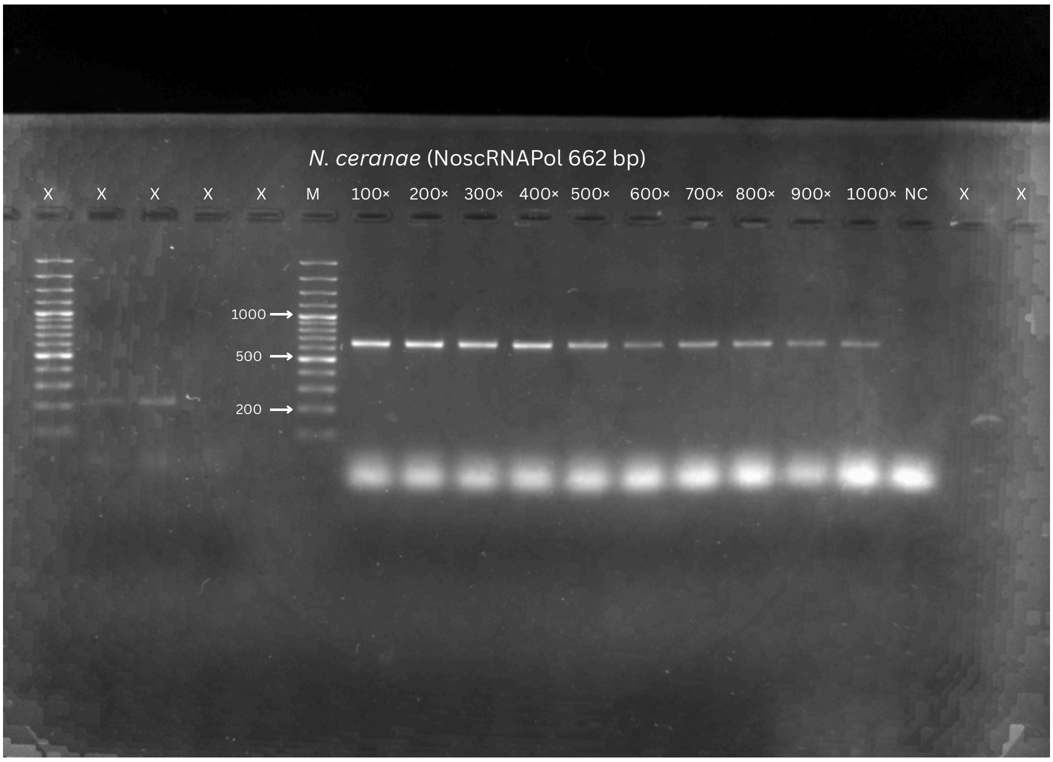

Figure S2-1A

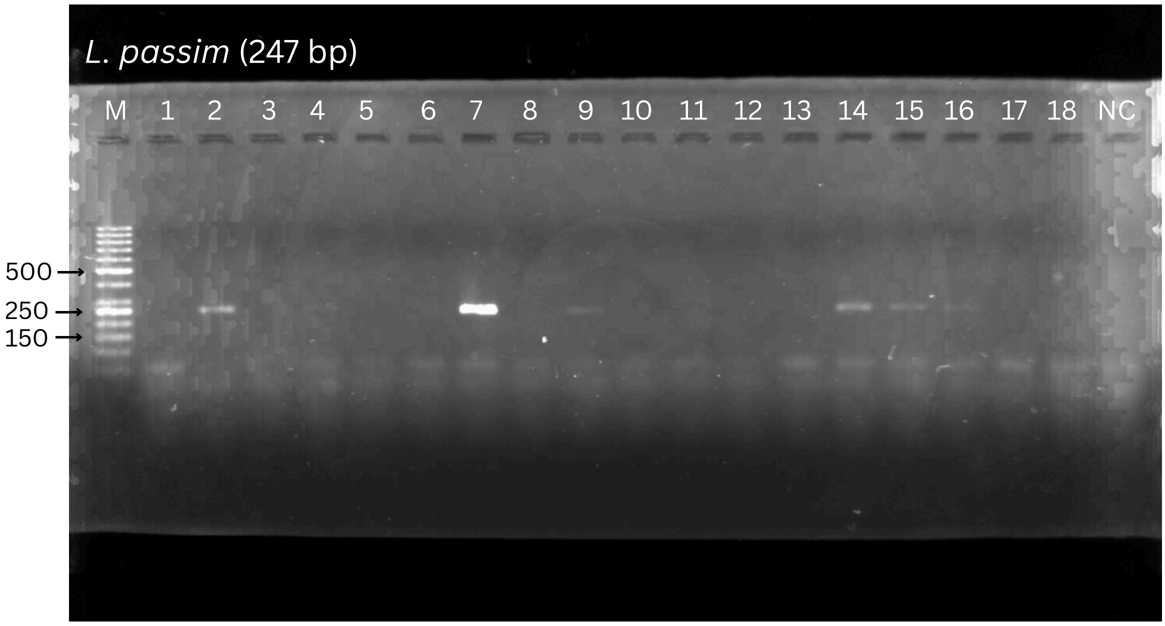

Figure S2-1B

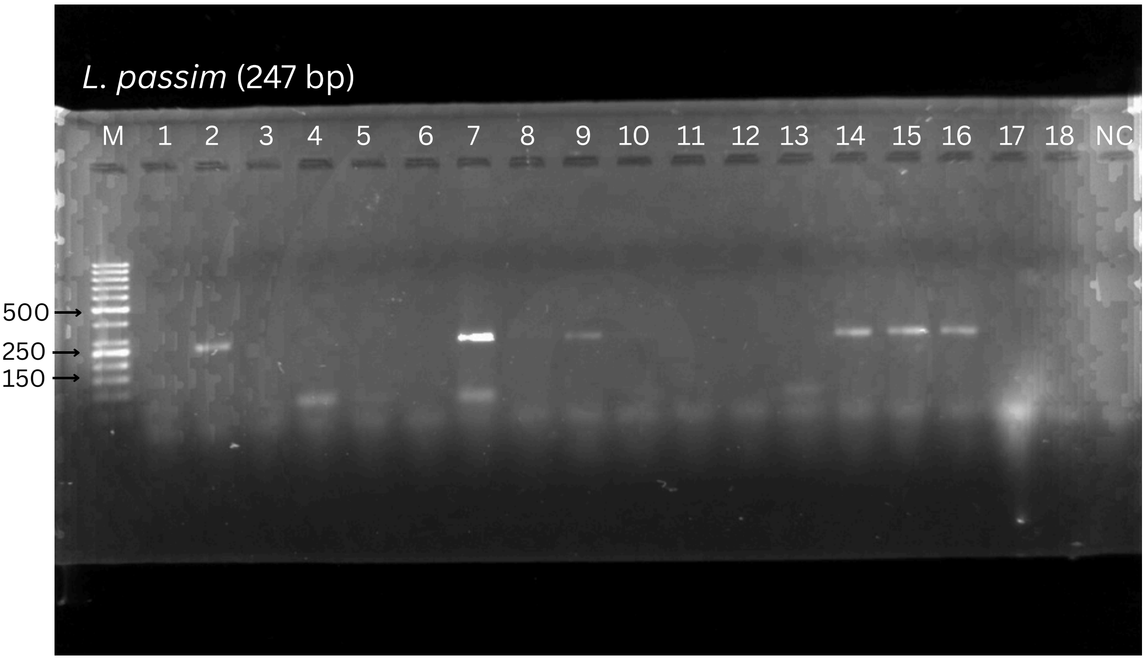

Figure S2-2A

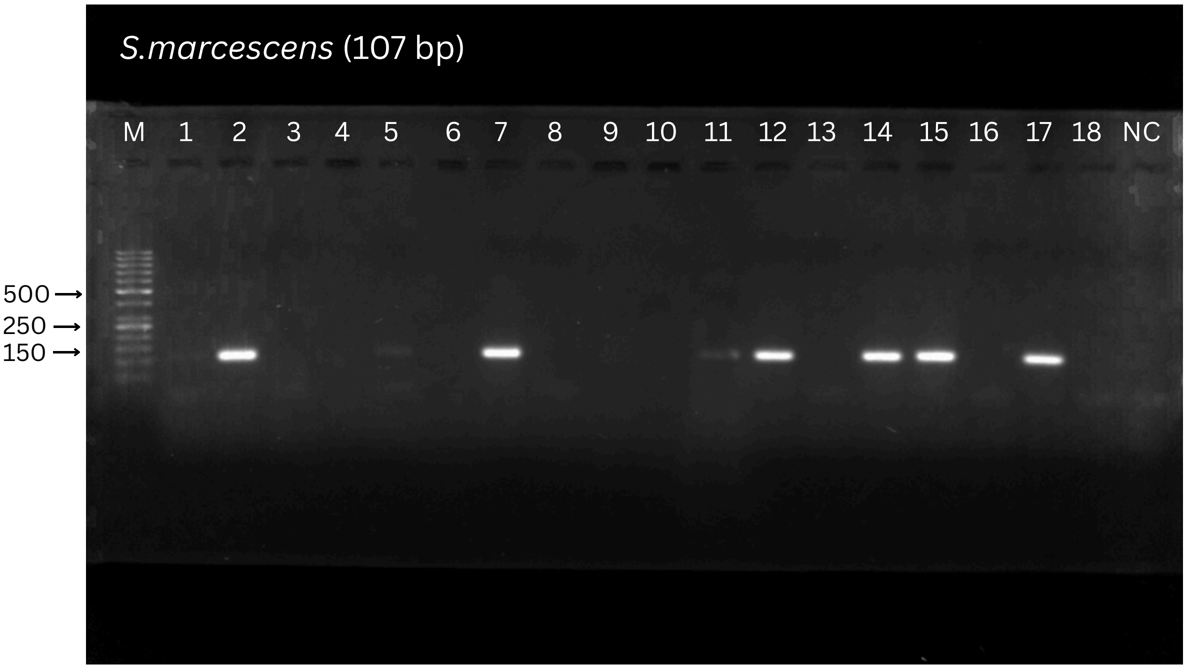

Figure S2-2B

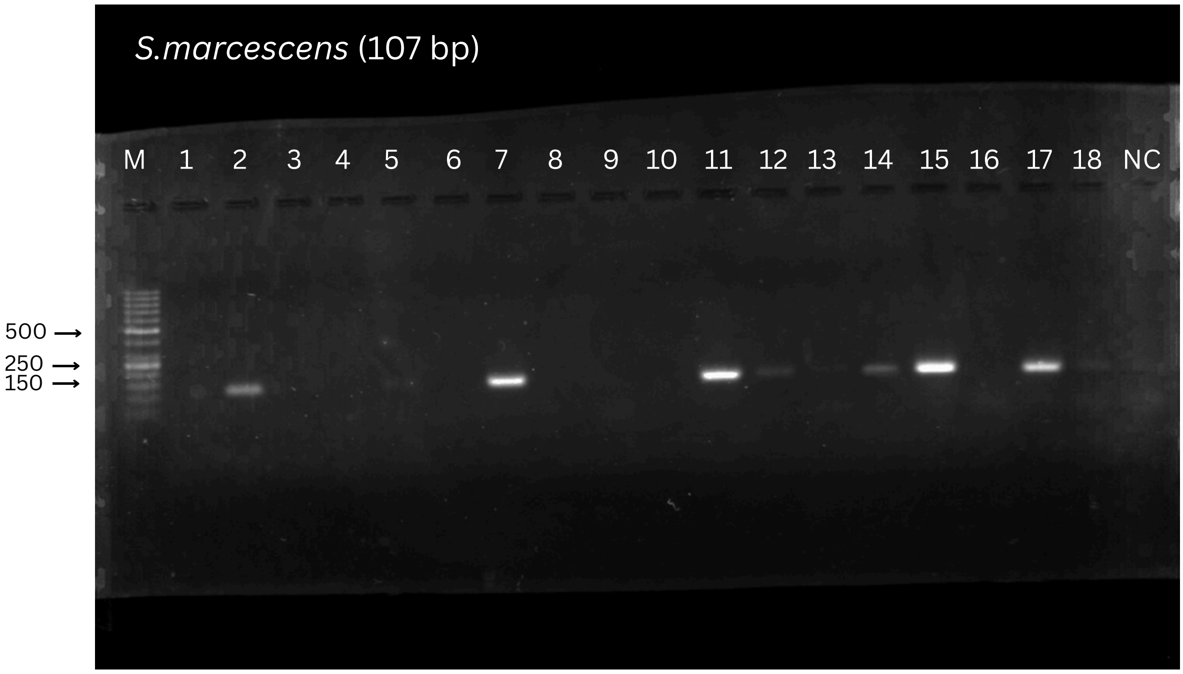

Figure S2-3A

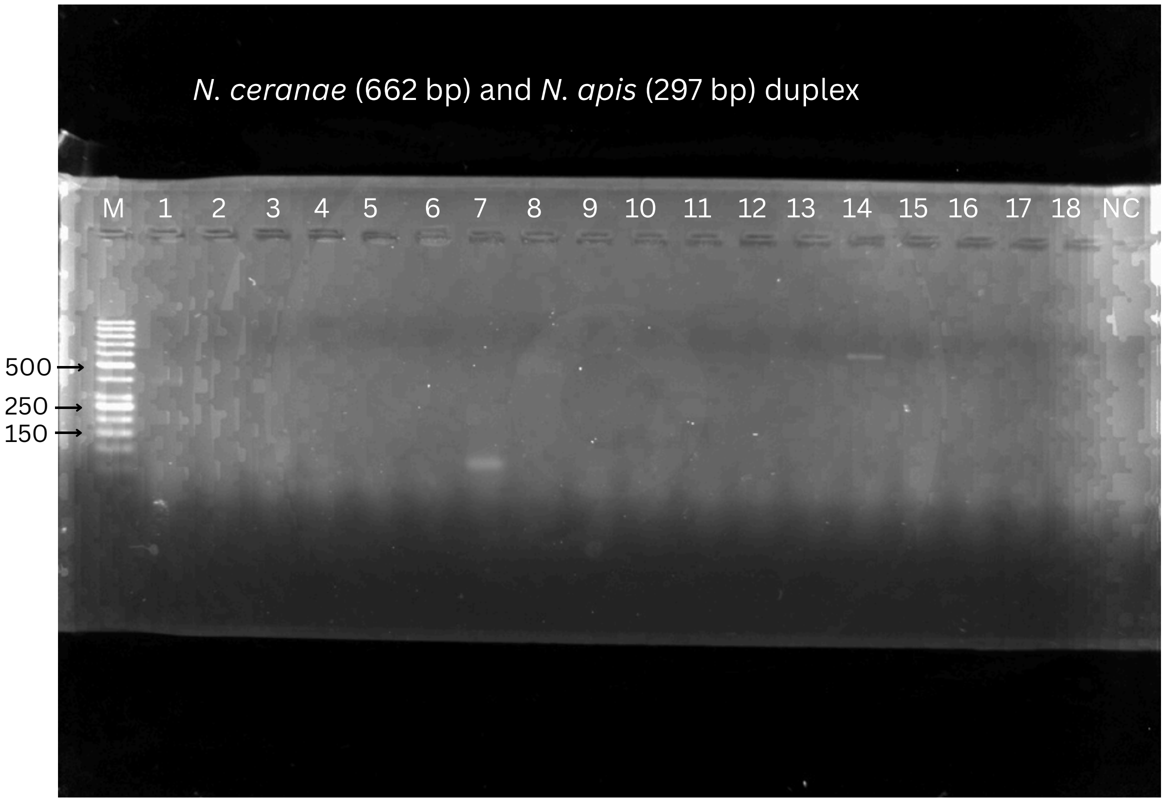

Figure S2-3B

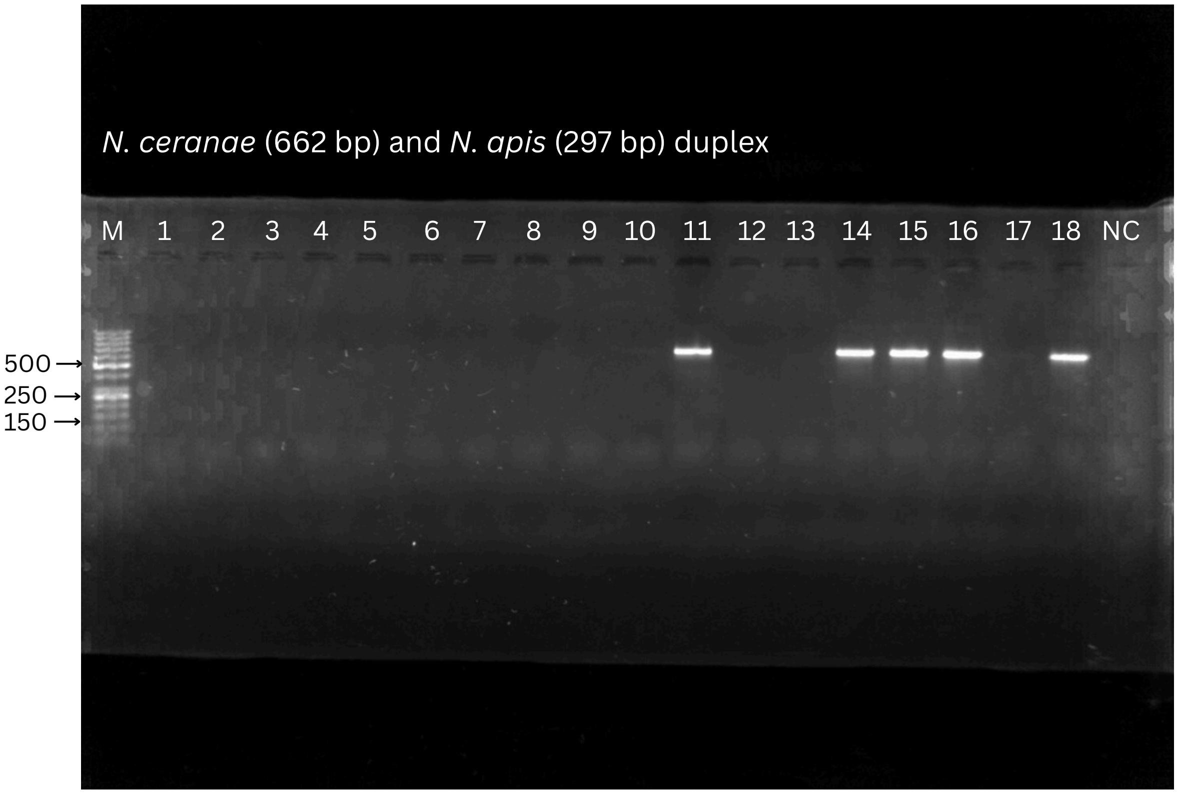

Figure S2-4A

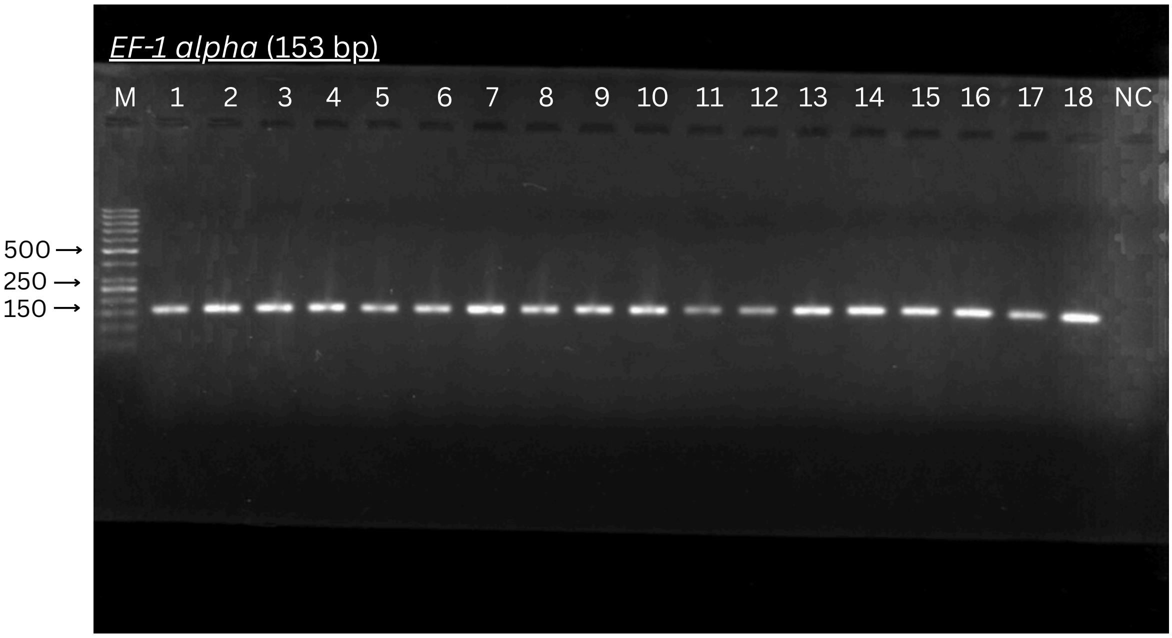

Figure S2-4B

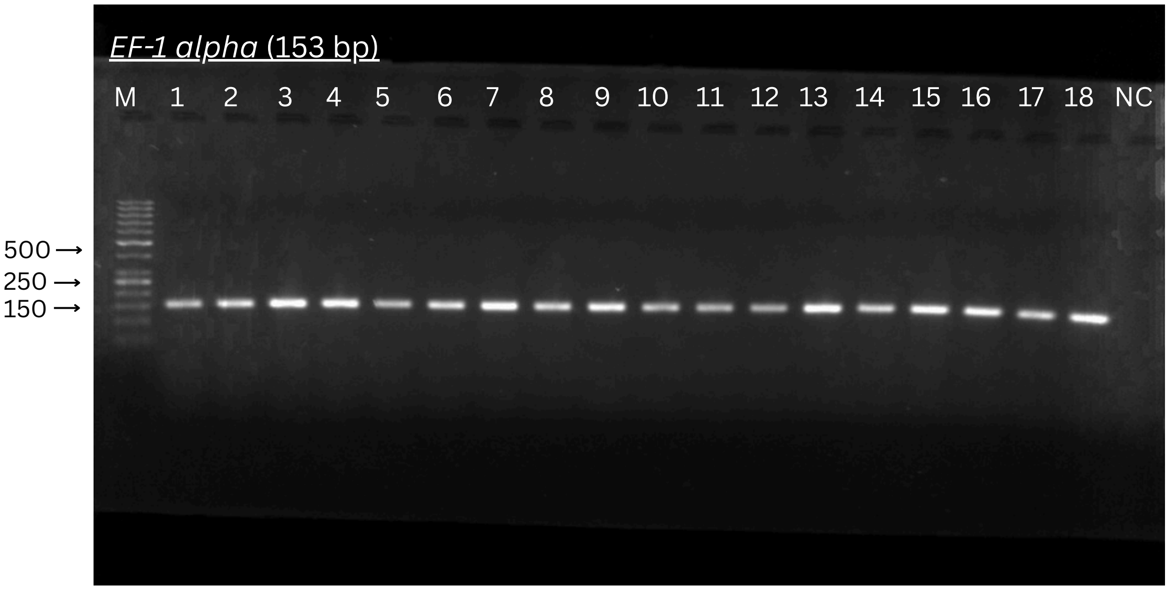
EF-1 alpha (153 bp)
M 1 2 3 4 5 6 7 8 9 10 11 12 13 14 15 16 17 18 NC
500
250
150

Figure S2-5A

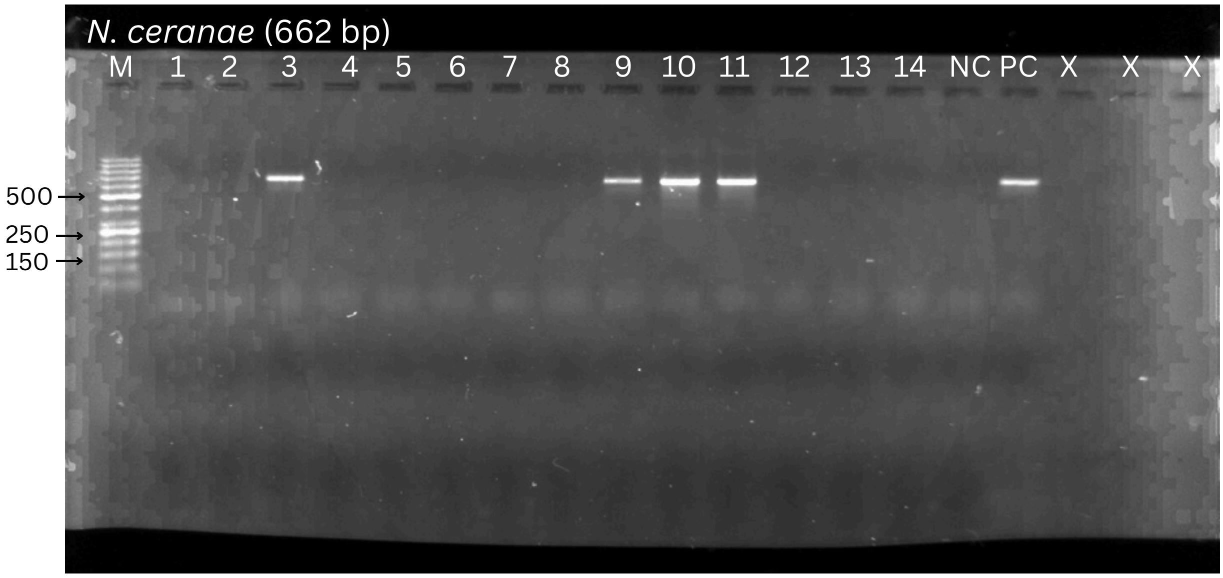

Figure S2-5B

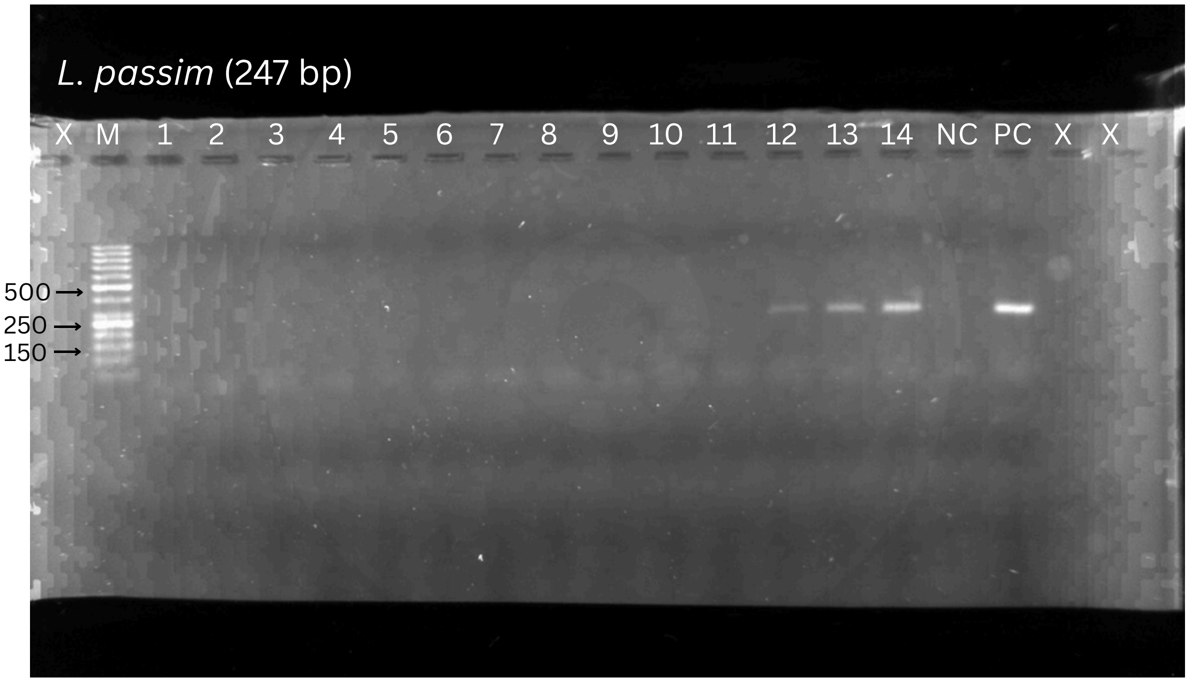

Figure S2-5C

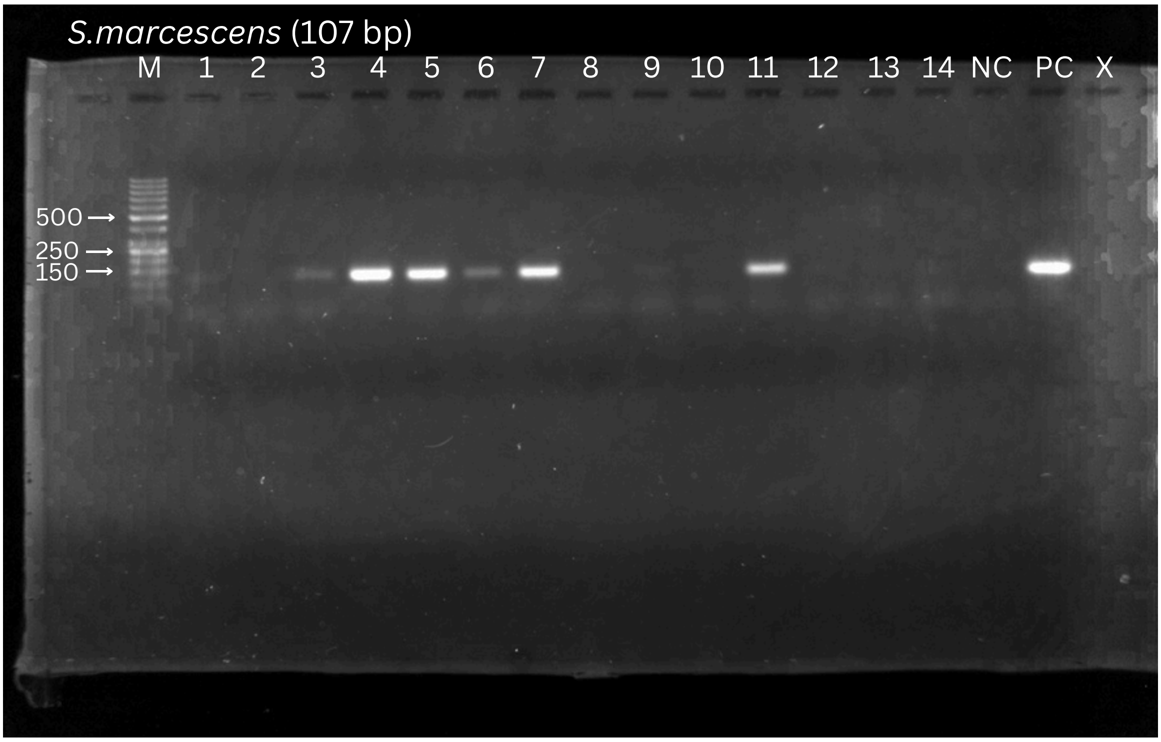

Figure S2-5D

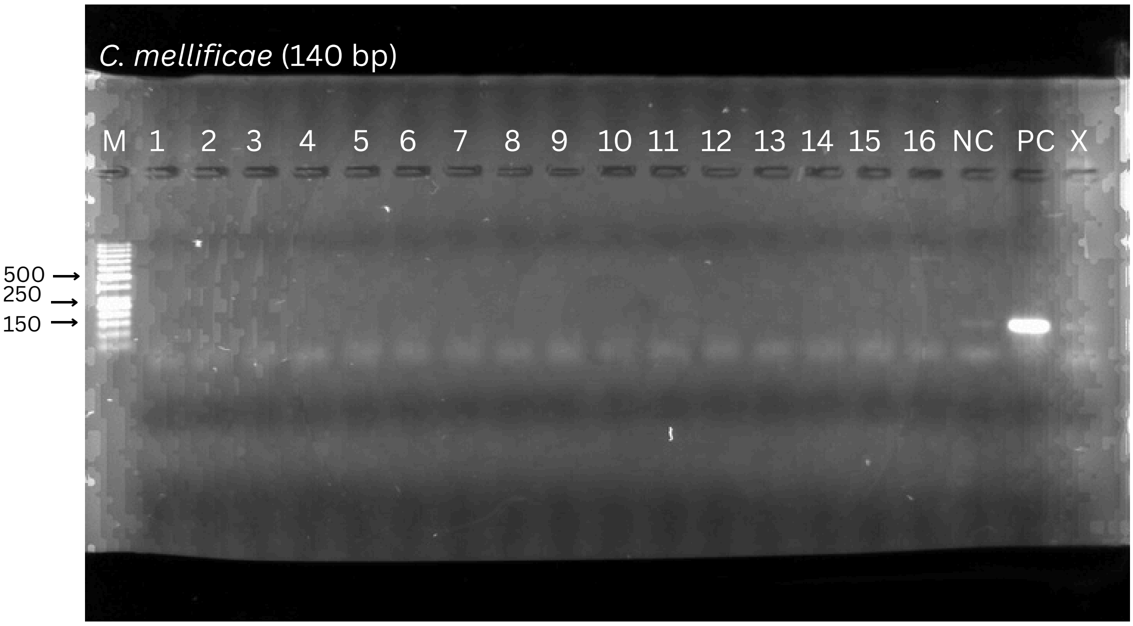

Supplement: S4 File — (PDF) [file pone.0334066.s004.pdf]
